# Unraveling the role of toxin-antitoxin systems in *Burkholderia pseudomallei*: exploring bacterial pathogenesis and interactions within the HigBA families

**DOI:** 10.1128/spectrum.00748-24

**Published:** 2024-06-25

**Authors:** Itziar Chapartegui-González, Jacob L. Stockton, Sarah Bowser, Alexander J. Badten, Alfredo G. Torres

**Affiliations:** 1 Department of Microbiology and Immunology, University of Texas Medical Branch, Galveston, Texas, USA; 2 Institute for Translational Sciences, University of Texas Medical Branch, Galveston, Texas, USA; 3 Department of Pathology, University of Texas Medical Branch, Galveston, Texas, USA; Universidad Andres Bello, Santiago, Chile

**Keywords:** *Burkholderia pseudomallei*, toxin-antitoxin, HigBA, persistence, antibiotics

## Abstract

*Burkholderia pseudomallei* (*Bpm*) is a Gram-negative intracellular pathogen that causes melioidosis in humans, a neglected, underreported, and lethal disease that can reach a fatal outcome in over 50% of the cases. It can produce both acute and chronic infections, the latter being particularly challenging to eliminate because of the intracellular life cycle of the bacteria and its ability to generate a “persister” dormant state. The molecular mechanism that allows the switch between growing and persister phenotypes is not well understood but it is hypothesized to be due at least in part to the participation of toxin-antitoxin (TA) systems. We have previously studied the link between one of those systems (defined as HigBA) with specific expression patterns associated with levofloxacin antibiotic exposure. Through *in silico* methods, we predicted the presence of another three pairs of genes encoding for additional putative HigBA systems. Therefore, our main goal was to establish which mechanisms are conserved as well as which pathways are specific among different *Bpm* TA systems from the same family. We hypothesize that the high prevalence, and sometimes even redundancy of these systems in the *Bpm* chromosomes indicates that they can interact with each other and not function as only individual systems, as it was traditionally thought, and might be playing an undefined role in *Bpm* lifecycle. Here, we show that both the toxin and the antitoxin of the different systems contribute to bacterial survival and that toxins from the same family can have a cumulative effect under environmental stressful conditions.

**IMPORTANCE:**

Toxin-antitoxin (TA) systems play a significant role in bacterial persistence, a phenomenon where bacterial cells enter a dormant or slow-growing state to survive adverse conditions such as nutrient deprivation, antibiotic exposure, or host immune responses. By studying TA systems in *Burkholderia pseudomallei*, we can gain insights into how this pathogen survives and persists in the host environment, contributing to its virulence and ability to cause melioidosis chronic infections.

## INTRODUCTION

Human melioidosis is a bacterial disease responsible for over 89,000 deaths per year, though the actual burden is thought to be underestimated due to different issues with adequate diagnosis (1). Melioidosis is a multifaceted disease caused by the Gram-negative pathogen *Burkholderia pseudomallei* (*Bpm*), a CDC Tier 1 Select Agent, that causes a myriad of symptoms that range from pneumonia to septic shock (2, 3). Both diagnosis and treatment are challenging because of the complex multidrug resistance phenotype and intracellular life cycle of the pathogen, which can cause both acute and chronic (also known as latent) infections, with an eradication treatment phase that lasts up to 12 months (1, 3, 4). An important aspect of the virulence and the study of *B. pseudomallei* is its large genome composed of two chromosomes that are over 7 Mbp in combined size. This relatively large genome harbors many paralogous genes with redundant functions, allowing *Bpm* to easily adapt to different environments without affecting virulent features (5, 6). All of this together makes it difficult to unravel the functional roles of some of those redundant gene products (3, 6–9).

Even with the appropriate diagnosis and treatment, clearance of the infection could be further complicated by the ability of *Bpm* to become persistent (10). The persistence phenotype, observed in a subpopulation of bacteria, is described as a dormant state that allows the microorganisms to survive various stressful stimuli (i.e., antimicrobial treatment and phagocytosis) (11, 12). It is unclear what regulatory pathways control this phenotype or what are the exact stimuli that trigger it, though some mechanisms have been proposed (13). Among the described molecular mechanisms for this phenotypic switch, occurring between the growing state and the dormant bacteria, the toxin-antitoxin (TA) systems have been implicated as potential regulators of persistence and their roles elucidated in other recalcitrant or chronic infections like tuberculosis (14). Toxin-antitoxin systems are small genetic elements widely distributed among prokaryotes whose products interact with each other to participate in many cellular processes (14). Their high prevalence in nature is also thought to be linked with the bacterial ability to adapt to unfavorable niches, environments, and/or conditions (14). These TA systems are classified based on their structure and mechanism of regulation, with seven different families currently described (15); however, types I through IV are the most studied (14). Among the seven families, type II TA systems are the most abundant and hence the best characterized. In the type II TA systems, the products of both genes are proteins that usually either form a protein-protein complex that keeps the toxin inactive, or the antitoxin can physically interact with the toxin’s target to block its function (14). Traditionally, most studies have focused only on the toxins, though more recently, there has been an increased number of publications showing that some antitoxins have their own self-secondary promoter (one that co-transcribes both the toxin and antitoxin and a second one that only transcribes the antitoxin) and have an independent function as transcriptional regulators (16–18). This is the case for the type II HigBA TA systems, which belong to the RelE/ParE superfamily.

The HigBA system and its double promoter regulation were first described on a plasmid (19, 20). Since then, several HigBA systems have been found in both plasmids and chromosomes, sharing the double promoter and negative regulation with the co-transcription from the antitoxin HigA (21). The antitoxin HigA expression has already been linked in some species with the expression of virulence factors, independently of the expression of the cognate toxin HigB (21). This system is peculiar not only because it has two promoters, but also because the *higB* toxin gene is located upstream of the antitoxin gene *higA* (22). It was generally believed that regulation of this kind of system is based on the theory that the antitoxin protein is less stable than the toxin, so it degrades easily under stressful conditions leading to the release and activation of the toxin (23). However, as our understanding of these systems has improved, this hypothesis was proved not valid for all of them, nor its degradation by Clp or Lon family proteases, as it was originally believed (13, 22, 23).

However, the roles of type II systems are still poorly understood and could likely have overlapping functions (24). Moreover, it has also been demonstrated that the components of some TA systems can sometimes interact with other TA systems, if the circumstances allow it, because they carry a conserved domain, with antitoxins being able to modulate toxins of different systems (24, 25). Among all of the predicted functions for the TA systems, only a few have been experimentally determined: post-segregational killing for plasmid maintenance (26), abortive infection to prevent phage replication (27), and persister formation for environmental stress tolerance (28).

We have previously analyzed the role of one of the predicted HigBA systems from *Bpm* (BPSL3343-BPS_RS18025)*,* concluding that the most important stimulus in order to function was the presence of the antibiotic levofloxacin (9). To unravel if the phenotypes associated with this putative HigBA function are shared in other TA systems from the same family and whether cumulative effects could exist, we analyzed the role of another predicted HigBA system encoded by the operon BPSL3260-BPSL3261. This work aims to describe the phenotypic and transcriptomic effect of the putative HigBA system encoded by BPSL3260-BPSL3261 as well as the one encoded by BPSL3343-BPS_RS18025 on *B. pseudomallei* K96243. Therefore, our main goal was to establish which mechanisms are conserved as well as which pathways are specific among different *Bpm* TA systems from the same family. We conclude that a clear relationship between the HigB toxin and the bacterial exposure to levofloxacin was found both *in vitro* and *in vivo*, showing a cumulative effect when two toxins from the same family (BPSL3261 and BPSL3343) were deleted together. We were also able to show that toxin-antitoxin systems do not function alone, but as part of a network and that various components of the different modules participate in bacterial homeostasis under different, and specific conditions. Although we are contributing with new insight into the functions of some toxin-antitoxin systems in *Bpm*, further studies are required to reach a comprehensive understanding of the complexities and the intricate network connections between these TA modules and their impact on bacterial survival and persistence.

## MATERIALS AND METHODS

### Bacterial strains and growth conditions

The bacterial strains used in this study are described in Table 1. *Bpm* strain K96243 was obtained from BEI Resources, Manassas, VA, USA. *Bpm* and *Escherichia coli* strains were routinely cultured on LB agar plates at 37°C, and antibiotics were supplemented as needed for selection (kanamycin and polymyxin for mutant construction, ampicillin for overexpression strains). Bacterial culture stocks were preserved in 20% glycerol (vol/vol) and stored at −80°C.

**TABLE 1:** Strains and plasmids used in the study

Strains	Characteristics	Reference
*B. pseudomallei* K96243 (WT)	BEI Resources (Manassas, VA, USA)	(5)
*B. pseudomallei* ICG001	*Bpm* K96243 ∆*higB*	(9)
*B. pseudomallei* ICG002	*Bpm* K96243 ∆*higB* ∆*higA*	(9)
*B. pseudomallei* ICG003	*Bpm* K96243 ∆BPSL3260	This publication
*B. pseudomallei* ICG004	*Bpm* K96243 ∆BPSL3261	This publication
*B. pseudomallei* ICG005	*Bpm* K96243 ∆BPSL3260 ∆BPSL3261	This publication
*B. pseudomallei* ICG006	*Bpm* K96243 ∆BPSL3260 ∆*higB*	This publication
*B. pseudomallei* ICG007	*Bpm* K96243 ∆BPSL3260 ∆BPSL3261 ∆*higB*	This publication
*E. coli* S17 λ*pir* pMo130-BPSL3261	BPSL3261 cloned into pMo130	This publication
*E. coli* S17 λ*pir* pMo130-BPSL3260	BPSL3260 cloned into pMo130	This publication
*E. coli* S17 λ*pir* pMo130-BPSL3261 (double)	BPSL3261 (double) cloned into pMo130	This publication
*E. coli* DH10 pBAD	Inducible vector	(29)
*E. coli* DH10 pBAD-BPSL3260	BPSL3260 in pBAD	This publication
*E. coli* DH10 pBAD-BPSL3261	BPSL3261 in pBAD	This publication
*E. coli* DH10 pBAD-BPSL3260-BPSL3261	BPSL3260-BPSL3261 in pBAD	This publication

### *In silico* and bioinformatics analysis

Classification of the previously predicted toxin-antitoxin systems (9) was compiled through different databases based on nucleotide and amino acid sequences (Genbank, Blast, and the *Burkholderia* Genome Database -www.burkholderia.com-) and 3D protein prediction [https://swissmodel.expasy.org; (30)]. However, none of them are canonically annotated as HigBA but are predicted for structure similarity and the specific genomic rearrangement with the toxin upstream of the antitoxin. Clustal Omega version 1.2.4 (https://www.ebi.ac.uk/Tools/msa/clustalo/) was used for sequence alignment (31). To predict the protein-protein interaction from those putative HigBA systems of *Bpm* K96243, STRING database version 11.5 [https://string-db.org; (32)] was used with the default setting of medium confidence required score (0.4), and the addition of extra nodes to each network once. The constructed networks were automatically clustered with MCL inflation parameter = 3 (accessed June 2023). To predict the relationship of those systems with the presence of prophage in the chromosome, the PHASTER software was used (https://phaster.ca, accessed June 2023) (33) with the *Bpm* K96243 genome [accession numbers: NC_006350.1 (chromosome 1) and NC_006351.1 (chromosome 2)].

### RNA extraction and cDNA synthesis

RNA from antibiotic-treated surviving bacteria was extracted using the Direct-zol RNA Miniprep Kit (Zymo Research), according to the manufacturer’s instructions. RNA concentration and purity were measured using an Epoch microplate spectrophotometer (Biotek) and stored at −80°C for future use. For intracellular bacteria, after cell permeabilization, two steps of differential centrifugation were used to enhance the ratio of bacterial RNA over the eukaryotic RNA (9, 34). RNA samples were used for RNA-seq analysis (Azenta, NJ, USA) or RT-qPCR.

### Expression quantification by RT-PCR analysis

The different RNA samples were used for cDNA synthesis using iScript cDNA Synthesis Kit (Bio-Rad), following the manufacturer’s instructions, with the subsequent conditions: 25°C for 5 min, 42°C for 30 min, 85°C for 5 min, 25°C for 5 min, 12°C for 5 min, 4°C store (9). The cDNA concentration and purity were measured, and samples were stored at −20°C for further use. Quantitative PCR (qPCR) was used to analyze the expression of the predicted HigBA toxin-antitoxin genes (BPSL3260-BPSL3261, BPSL0174-BPSL0175, and BPSS1061-BPSS1060) for surviving bacteria after antibiotic treatment or intracellular survival between the ∆BPSL3343 (also named, ∆*higB*) and the wild-type (WT) K96243 or the ∆BPSL3343 ∆BPS_RS18025 (also named, ∆*higB* ∆*higA*) and the WT, to see the role of the redundant systems under the different conditions.

Gene expression quantification was performed using QuantiNova SYBR Green (Qiagen) following the manufacturer’s instructions, using the WT expression as the control for expression in each condition, in a Bio-Rad CFX96 thermocycler. Expression was normalized with the housekeeping genes *rpoB* and 16S for each condition, following the ΔΔ*C*_*t*_ method (35). All primers used for expression are summarized in Table 2 in a final concentration of 0.7 µM each. As recommended, the template cDNA added per reaction was 100 ng. The PCR cycling program was set as follows: initial heat activation step 95°C 2 min; two-step 40 cycles of 5 s 95°C and 30 s 60°C. The threshold cycle (*C*_*t*_) and melting curve of each gene were automatically established and recorded by the software CFX Maestro Software (version 4.0). Two-way ANOVA analysis followed by Turkey’s multiple comparisons test was conducted to establish significant differences among strains in the different conditions, and significance was considered *P* < 0.05.

**TABLE 2:** List of primers designed and used in this study

Primer	Sequence	Experiment
F1F_3260	GAGCTGATATCAGGGCCCCGAGCTAAGGATCTATCTGAG	Mutant construction
F1R_3260	GCTCTACGTGGATGATTTTTCATGACCATG	
F2F_3260	AAAAATCATCCACGTAGAGCCTGAGCTG	
F2R_3260*	GATTAATTGTCAACAGCTCAGCATTGCCCCTCGTGATG	
F1F_3261	GAGCTGATATCAGGGCCCCGGCAGCCGGTGCGGATACG	
F1R_3261	CAACTGTTCCCGAGCTGCATTGCCGTGTG	
F2F_3261	ATGCAGCTCGGGAACAGTTGAAGTGCCATGGC	
F2R_3261	GATTAATTGTCAACAGCTCAGGTCATCCTGACGCGCGC	
F1F_3260 (double)	GAGCTGATATCAGGGCCCCGAAAAGCGCACCGGCCGGC	
F1R_3260 (double)	GCTCTACGTGTCGCCGGGGTGCGGATGATTTTTC	
F2F_3260 (double)	ACCCCGGCGACACGTAGAGCCTGAGCTG	
F2R_3260 (double)*	GATTAATTGTCAACAGCTCAGCATTGCCCCTCGTGATG	
BPSL0175 F	TCGGATCGAACTCATCATGC	qPCR
BPSL0175 R	CCTTCCATGTCGACACCTG	
BPSL0174 F	GAATGTCACCGCGAGTCTT	
BPSL0174 R	CTGGAGTCGATGTGTGATCTG	
BPSL3261 F	CCTCTTTGTGGTCGTCTTCTATC	
BPSL3261 R	ACTTCAACTGTTCCGCATACT	
BPSL3260 F	GACCTTGCAGAGCAACTATGA	
BPSL3260 R	ATCCACCTCACCATGCAAAT	
BPSS1060 F	CATGGACGAGATACCGAGATG	
BPSS1060 R	GAAACGTGAGGAATACGCAATAG	
BPSS1061 F	TTCGACGGTGTTTCTGATCG	
BPSS1061 R	GCGGCACACGTCGTATT	
16S F	GCGTAGAGATGTGGAGGAATAC	
16S R	ACCAGGGTATCTAATCCTGTTTG	
*rpoB* F	CCGAAGGACGTGCTGTATTT	
*rpoB* R	GTGAAGTTGTCGAAGACGAAGA	
pBAD-3260 F	ATTAACCATGGATCCGAGCTATGGTCATGAAAAATCATCCGC	Overexpression
pBAD-3260 R	TTCGAATTCCCATATGGTACTATTTGTCGTGGCCCGGG	
pBAD-3261 F	ATTAACCATGGATCCGAGCTATGGCAACCATGCACGATAC	
pBAD-3261 R	TTCGAATTCCCATATGGTACTCATGACCATGCCTCGTTAG	
pBAD-3260–3261 F	ATTAACCATGGATCCGAGCTATGGCAACCATGCACGATAC	
pBAD-3260–3261 R	TTCGAATTCCCATATGGTACCTATTTGTCGTGGCCCGG	

^*a*^ F_ depicted forward primer and R_ the reverse primer. Each gene is depicted with its numeral designation.

### Mutant construction

The single (ΔBPSL3260, named ICG003; ΔBPSL3261, named ICG004), double (ΔBPSL3260ΔBPSL3261, named ICG005; ΔBPSL3261 ΔBPSL3343, ΔBPSL3261 Δ*higB*, named ICG006), and triple (ΔBPSL3260 ΔBPSL3261 ΔBPSL3343, ΔBPSL3260 ΔBPSL3261 Δ*higB*, named ICG007) mutant strains were constructed using a biparental matting approach for allelic exchange using the plasmid pMo130, as previously described (36). All the primers used are listed in Table 2. Briefly, pMo130 was digested and linearized with Hind*III*-HF (NEB) and Nhe*I*-HF (NEB) following the established protocol (37). Both upstream and downstream fragments of 400–600 bp from the target gene were amplified with Q5 High-fidelity DNA polymerase (NEB), containing the initial and final sequence of each gene. Assembly of the three fragments was constructed using Gibson Assembly kit (NEB) and transformed into *E. coli* S17-1 λ*pir*. The plasmids carrying intragenic *in-frame* deletions were confirmed by PCR sequencing in the University of Texas Medical Branch (UTMB) Sequencing Facility. The donor strain for allelic exchange *E. coli* S17-1 λ*pir*, with recipient *Bpm* through biparental matting, was used. For the construction of the single mutant strains, *Bpm* K96243 wild type was used for the matting; for the double mutants’ construction, the ΔBPSL3261 strain (ICG004) was used; and for the triple mutant, the ΔBPSL3261 ΔBPSL3343 strain (ICG006) was employed. Merodiploids were selected by supplementing kanamycin (500 µg/mL) and polymyxin B (30 µg/mL) into LB agar plates. Colonies that turned yellow after pyrocatechol exposure were selected as positive for the deletions and they were counter-selected on YT agar supplemented with 15% sucrose. The intragenic deletions from the selected clones were confirmed by PCR sequencing (UTMB Sequencing Facility). Because *Bpm* is a Tier 1 Selected Agent, complementation of the mutants was not performed to avoid gain of function or enhance virulence phenotypes.

### Bacterial growth curves

Growth curves were conducted to confirm that fitness differences among the strains were not present. Isolates from overnight cultures were inoculated in 30 mL of nutrient-rich medium LB, minimal medium M9, or host-like medium Dulbecco’s modified Eagle’s medium (DMEM) and incubated at 37°C for 24 h with shaking at 200 rpm. The optical density (OD_600_) and colony-forming units (CFUs) enumeration were measured and validated from each time point. The experiments were performed in triplicate. Multiple unpaired *t* test analysis was carried out to establish significant differences among mutant strains and WT in the different media and significance was considered *P* < 0.05.

### Antibiotic-induced persistence assays

The quantification of the persister frequency or the survival population after antibiotic treatment was performed as previously described (9). Briefly, wild-type and mutant strains were grown overnight for 12 h in LB with agitation at 200 rpm at 37°C and the cultures were inoculated at 1 × 10^8^ CFU/mL in LB containing 100× minimum inhibitory concentration (MIC) of levofloxacin, ciprofloxacin, ceftazidime, meropenem, or doxycycline and further incubated in static conditions at 37°C for 24 h. Bacteria inoculated in LB without antibiotics were used as a control. Bacteria were serially diluted for CFU enumeration after 24 h and the survival rates were quantified with the input numbers. All assays were performed in triplicate. One-way and two-way ANOVA followed by Dunnett’s multiple comparison tests were used to establish statistical differences, and significance was considered *P* < 0.05.

### Killing curves

Killing curves in presence of fluoroquinolones were performed as previously described (9) in the *Bpm* WT K96243 strain and all the isogenic mutants created for this work. Briefly, overnight cultures were subculture in 20 mL of LB broth, at a starting concentration of 1 × 10^6^ CFU/mL, which was supplemented with both fluoroquinolones (levofloxacin and ciprofloxacin) at supra-lethal concentrations. To establish the killing rates of the antimicrobials on the different strains, viability was assessed by CFU enumeration up to 12 h of incubation at 37°C with agitation. Multiple unpaired *t* test were used to establish statistical differences, and significance was considered *P* < 0.05 across three independent replicates.

### Biofilms formation in antibiotic-induced persister cells

Biofilm formation capacity was evaluated in Costar 24-well plates (Corning). The strains were grown overnight in LB for 12 h and used to inoculate at 1 × 10^8^ CFU/mL in LB alone or supplemented with 100× MIC of levofloxacin, ciprofloxacin, ceftazidime, meropenem, or doxycycline. Plates were statically incubated for 24 h at 37°C. After incubation, bacteria were serially diluted for CFU enumeration for biofilm normalization with output numbers as previously described (38). After that, the wells were washed three times with water and stained with 1% crystal violet (wt/vol) for 30 min. The stained biofilms were solubilized with 1 mL of 35% acetic acid (vol/vol). Biofilm formation was quantified by measuring the optical density at 600 nm, using non-inoculated wells containing LB as blank. One-way ANOVA followed by Dunnett’s multiple comparison test was used to establish statistical differences, and significance was considered *P* < 0.05.

To evaluate the biofilm formation by intracellular bacteria, the cells recovered from macrophages were used to inoculate fresh LB and incubated and stained as described above. The adherence capacity was compared among the intracellular bacteria in macrophages treated with kanamycin to kill extracellular bacteria for 6 h and bacteria from macrophages treated with levofloxacin for 12 and 24 h. Plates were incubated statically for 24 or 48 h if there was no growth since persisters need a longer time to recover to regular growing rates. Two-way ANOVA followed by Dunnett’s multiple comparisons was used to establish statistical differences, and significance was considered *P* < 0.05.

### Kill-rescue and overexpression assays in inducible systems

The inducible vector pBAD (ThermoFisher Scientific) maintained in *E. coli* DH10 (NEB) was used for the gene overexpression. The toxin (BPSL3261), antitoxin (BPSL3260), or both toxin-antitoxin (BPSL3260-BPSL3261) genes were amplified with Q5 High-fidelity DNA polymerase (NEB) using the primers listed in Table 2; the vector was digested with Kpn*I*-HF and Sac*I*-HF (NEB); and they were assembled using Gibson Assembly kit (NEB). Selected plasmid sequences were confirmed by sequencing at the UTMB Core Sequencing Facility.

The impact of gene induction expression on growth was evaluated independently by two methods, “kill-rescue” (39) and traditional “overexpression” (9) assays. Such methods were performed to also evaluate the effect of both short- and long-term inductions on bacteria growth. In both cases, media were supplemented with ampicillin for plasmid maintenance, arabinose was added for induction, and the empty vector (pBAD) was used as a growing control.

For kill-rescue curves, the overnight cultures from all the strains [empty vector (pBAD), vector with the toxin (pBAD-BPSL3261), vector with the antitoxin (pBAD-BPSL3260), or vector with the full system (pBAD-BPSL3260-BPSL3261) inserted] were 1:500 diluted in fresh LB supplemented with ampicillin and 0.2% glucose at 37°C until they reached OD_600_ ~0.1–0.2. After that, cultures were pelleted and resuspended in fresh supplemented-ampicillin LB, with or without inducer (0.2% arabinose final concentration). Cultures were then inoculated onto a 96-well plate, incubated at 37°C with shaking, and the OD_600_ was measured every 15 min.

The overexpression assays were carried out in LB and minimal M9 media supplemented with ampicillin. The four constructs were cultured overnight for 12 h and diluted in 100 mL of fresh media supplemented with ampicillin. Gene expression was induced with 0.2% arabinose when the culture reached OD_600_ ~0.2 in M9 and OD_600_ ~0.5 in LB, and optical density was measured every hour for 8 h and at 24 h. Statistical unpaired *t* test analysis was carried out to establish significant differences between constructs and empty vector growth, and significance was considered *P* < 0.05.

### Macrophage uptake and intracellular survival assays

The intracellular survival of wild-type and mutant strains was evaluated using the murine macrophage cell line RAW 264.7 (TIB-71️). The cells were routinely grown in DMEM supplemented with 10% fetal bovine serum, 1 mM sodium pyruvate, 1 mM non-essential amino acids, and 1% penicillin-streptomycin. *Bpm* strains were cultured overnight for 12 h in LB at 37°C with 200 rpm shaking. The murine macrophages were seeded at 2 × 10^5^ cells/mL in Costar 24-well plates (Corning), and they were infected with the different strains at a multiplicity of infection (MOI) of 10 for 30 min (uptake step). After that, the supernatants were collected, the cells were washed two times with phosphate-buffered saline (PBS), and incubated with DMEM supplemented with 250 µg/mL kanamycin for extracellular bacterial killing. For bacterial enumeration, the cells were washed with PBS before lysing with 0.1% Triton X-100. The lysed cells were serially diluted and plated on LB agar (output). The uptake percentage was determined with the input bacterial number $\left(\%\;Uptake=\frac{CFU\;recovered\;after\;30\;min}{CFU\;input}\times 100\right)$, while the intracellular bacteria were normalized using the uptake concentration for each strain ($\%\;Survival=\frac{CFU\;output}{CFU\;uptake}\times 100$). All the assays were performed in triplicate.

Alternatively, to test the macrophage-induced persister phenotype, DMEM media were supplemented with 80 µg/mL of the cell-permeable antibiotic levofloxacin (40) after the 30-min incubation for the uptake step as previously described (37). Then, intracellular bacteria were collected at 12 and 24 h post-infection and plated for CFU enumeration. The surviving intracellular bacteria were normalized using the uptake concentration for each strain, as described above. All the assays were performed in triplicate. Alternatively, these bacteria were also used to evaluate the biofilm formation ability once exposed to fresh media. One and two-way ANOVA followed by Dunnett’s multiple comparison tests were used to establish statistical differences, and significance was considered *P* < 0.05.

### RNA-seq

For RNA-seq analysis, samples were directly submitted to Azenta (NJ, USA). RNA samples were quantified using a Qubit 2.0 Fluorometer (ThermoFisher Scientific) and RNA integrity was checked with 4200 TapeStation (Agilent Technologies). Samples were initially treated with TURBO DNase (ThermoFisher Scientific) to remove DNA contaminants. The rRNA depletion sequencing library was prepared by using QIAGEN FastSelect Bacteria rRNA HMR Kit (Qiagen). RNA sequencing library preparation used NEBNext Ultra II RNA Library Preparation Kit for Illumina by following the manufacturer’s recommendations (NEB). Sequencing libraries were validated using the Agilent TapeStation 4200 and quantified using Qubit 2.0 Fluorometer. The sequencing libraries were multiplexed and clustered on one flow cell and it was loaded on the Illumina Hiseq instrument according to the manufacturer’s instructions. The samples were sequenced using a 2 × 150 pair-end configuration, converted into fastq files, and de-multiplexed using Illumina bcl2fastq program v2.20. One mismatch was allowed for index sequence identification. After demultiplexing, sequence data were checked for overall quality and yield. Raw sequence reads were trimmed to remove possible adapter sequences and nucleotides with inferior quality using Trimmomatic v.0.36. The reads were then mapped to the *Bpm* reference genome available on ENSEMBL using the STAR aligner v.2.5b, generating BAM files. Unique gene hit counts were calculated by using feature Counts from Subread package v.1.5.2. Only unique reads that fell within exons were counted.

The trimmed results were submitted to RaNA-seq software, which was used to establish expression differences and results visualization (41). This software establishes differential expression analysis with DESeq2 (42), normalizing after the expression values as TPMs (transcripts per million) with a cutoff of 0.05 for results representation. In each sample, the first one hundred genes with the lowest *P* value are used for heatmap representation. The product of the coding retrieved by the software was identified through the database www.burkholderia.com (last accessed December 2023) or predicted through BLAST alignment against “Burkholderiales” when the database only identified them as “hypothetical protein.” Only the top 100 most significant differentially expressed genes were shown for each comparison.

### *In vivo* bacterial infection model

Female 6-to-8-week-old BABL/cJ mice were purchased from Jackson Laboratories (Bar Harbor, ME), and were acclimated for 7 days before experiments. Mice were housed in microisolator cages under pathogen-free conditions, maintained on a 12 h light cycle, and provided with rodent feed and water *ad libitum*. Anesthetized BALB/cJ mice (*n* = 5 per group) were intranasally (i.n.) inoculated with 50 µL (25 µL/nare) *Bpm* K96243 wild-type morphotype I or different mutant strains (ΔBPSL3260; ΔBPSL3261; ΔBPSL3260 ΔBPSL3261; ΔBPSL3261 Δ*higB*; and ΔBPSL3260 ΔBPSL3261 Δ*higB*) equivalent to ~3–3.5 LD_50_ (1 LD_50_ = 312 CFU, based on WT values). Mice were treated daily for 5 days with intraperitoneal (i.p.) injections of levofloxacin (25 mg/kg per day in PBS) starting at 24 h post-infection (pi). Animals were checked and weighed daily for up to 21 days pi since the animal model mimics a chronic infection. Humane endpoints were established (≤80% initial weight, disease symptoms, and/or distress) and strictly monitored every day as described in the animal protocol IACUC #0503014F approved by the Animal Care and Use Committee of the University of Texas Medical Branch. The organs of surviving mice were collected, homogenized, serially diluted, and plated onto LB for bacterial burden determination. Two-way ANOVA followed by Dunnett’s multiple comparison test was used to establish statistical differences, and significance was considered *P* < 0.05.

## RESULTS AND DISCUSSION

### *In silico* analysis and bioinformatics predictions establish a clear relationship between TA systems and mobile elements, revealing a network of direct protein-protein interactions

**Fig 1:**
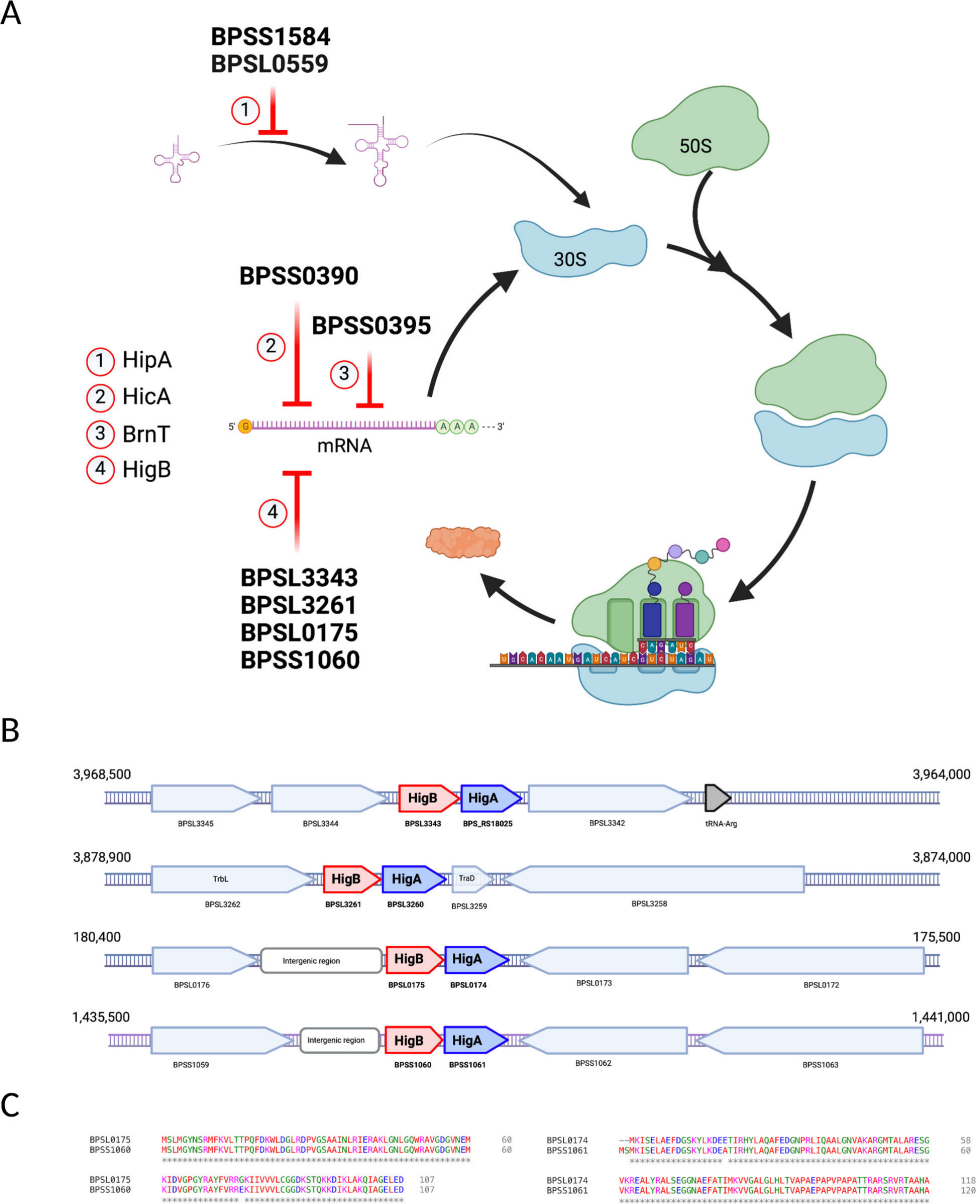
HigBA predicted systems in *Burkholderia pseudomallei* strain K96243. (**A**) Predicted toxin-antitoxin systems for which the toxin target is part of the protein synthesis. (**B**) Genomic environment and flanking genes of the four HigBA predicted systems. (**C**) Amino acid alignment (Clustal) that shows the high identity between the predicted systems encoded by BPSL0175-BPSL0174 and BPSS1060-BPSS1061. Images generated with BioRender.com.

*Bpm* is a human pathogen with a large genome composed of two chromosomes (1, 3, 43). Function prediction and genome annotation are not easy because most of the analysis focuses on virulence factors (i.e., secretion systems or adhesins) and also due to the different nomenclature used in different databases and among strains (44, 45). Previous work has established the presence of toxin-antitoxin systems in *Bpm* genomes and recognized a relationship between them and the induction of the persister phenotype. The identification and characterization of novel TA modules in human pathogens could enhance our understanding of the virulence traits, potentially leading to more effective and successful treatments. In the context of *Bpm*, this could translate into a reduction in relapse rates, shortened treatment durations, and improved overall outcomes. Numerous TA systems have been predicted by bioinformatics tools, but only a few have received experimental scrutiny (7–9, 37, 46). Hence, we identified the putative toxin-antitoxin systems (Fig. 1A) using different databases and sequence homology platforms. We also found that many of the putative toxins function at different steps of protein synthesis based on their predicted family, including the four identified as HigB endoribonuclease toxins (47). The HigBA system has been found in other human pathogens (17, 48, 49), with the toxin HigB being a ribosome-dependent endoribonuclease from the RelE toxins family that cleaves prokaryotic mRNA, and HigA acting as a transcriptional regulator (16, 21–23, 50).

Toxin-antitoxin systems have been extensively linked with mobile elements such as plasmids and phages (14, 22), and through *in silico* prediction we also found that three out of four predicted HigBA systems in *Bpm* were linked to integrated phages (Fig. S1). All of them shared similar genomic arrangements and flanking genes (Fig. 1B), enhancing the idea that these systems are encoded within highly mobile elements. Previously, we described the toxin encoded by the gene BPSL3343 (9) and in this study, our objective was to decipher the role of the system encoded by BPSL3260-BPSL3261. Because the other two predicted HigBA shared over 95% identity (Fig. 1C) and are part of the closely-related *Myoviridae* phages (51) (Fig. S1), integrated into different chromosomes (BPSL0174-BPSL0175 chromosome 1 [Fig. S1A]; BPSS1060-BPSS1061, chromosome 2 [Fig. S1B]), it is challenging to target only one of the systems to decipher their functions. This also correlates with the *in vitro* characterization of those toxins, due to the highly similar results observed by other authors (7). Hence, we focused this study on the above mentioned system (BPSL3260-BPSL3261).

Because the presence of some of those systems in high-mobile elements, we evaluated their presence and conservation among other *Bpm* strains, as well as the closely related *B. thailandensis,* through BLAST prediction, as it was previously performed for other predicted toxins (8). The BPSL3343 was present in 7 strains of *B. thailandensis*, and in 12 different *Bpm* strains, while not at 100% identity. Interestingly, the counteracting antitoxin BPS_RS18025 was not present in any of them. Regarding the BPSL0174-BPSL0175 and BPSS1060-BPSS1061, due to their high identity and to confirm their specific presence and eliminate cross-detection, we only considered the results that showed 100% identity. In that case, BPSL0174-BPSL0175 was present in four strains of *B. thailandensis* but not the BPSS1060-BPSS1061. However, the BPSS1060-BPSS1061 was present in two other *Bpm* strains (PHILS112 and HBPUB1013a). Though, the BPSL0174 and BPS0175 were not well conserved in *Bpm*, since 17 strains carried the gene for BPSL0174 (antitoxin) but only 8 for BPSL0175 (toxin), and only 1 out of them carried both genes (TSV48). The genes BPSL3260-BPSL3261 were present in one *B. thailandensis* strain (AW34-19p). Outside the *pseudomallei* complex, BPSL3260-BPSL3261 exhibited over 80% identity in genomic alignment with some *Burkholderia cenocepacia* sequenced strains, but no prediction was found outside the *Burkholderia* spp. The putative antitoxin BPSL3260 was also found in one strain of *Burkholderia multivorans*, but not the putative toxin BPSL3261.

**Fig 2:**
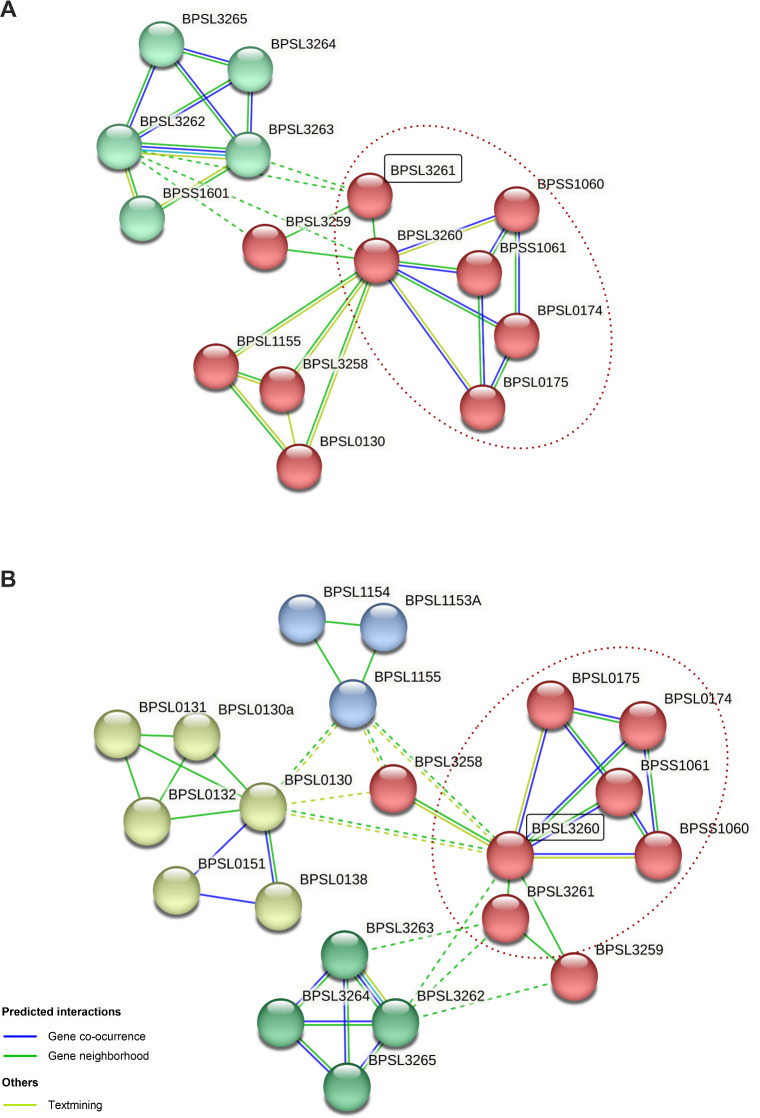
STRING network of the predicted system HigBA encoded by BPSL3260-BPSL3261 in *B. pseudomallei* K96243. Network generated with (A) the toxin BPSL3261 or (B) the antitoxin BPSL3260, as connection point among the pathways. In both cases, two out of three other HigBA predicted systems were connected to them by co-occurrence, as well as the cluster BPSL3262-BPSL3265 that encodes different plasmid conjugal transfer proteins. In the case of the toxin (**A**)*,* it is also connected to two hypothetical proteins (BPSL1155 -putative transcriptional regulator- and BPSL0130) and an ATPase (BPSL3258). In the case of the antitoxin (**B**)*,* the yellow cluster mostly encodes phage-like proteins (BPSL0130-BPSL0132), while the blue cluster encodes two hypothetical proteins (BPSL1154 and BPSL1153a) and a putative transcriptional regulator (BPSL1155). Red dashed lines circle the TA systems components; black squares indicate the origin of the network.

Recent evidence supports the notion that these TA systems are interconnected, particularly those belonging to the same family (25). To assess the interconnectivity network of these proteins, we used the STRING database for both the putative toxin BPSL3261 (Fig. 2A) and its corresponding antitoxin BPSL3260 (Fig. 2B). In both cases, there was a strong connection with some of the other TA systems (spotted in red), mostly based on co-occurrence. The same analysis was also performed with the other putative HigBA systems (Fig. S2A and C, toxins; B, antitoxins), and they showed not only a relationship with the other systems from the same family but also with other TA systems like BPSS0395-BPSS0394, predicted as BrnT/BrnA, highlighting the complex network existing within those systems. Moreover, both components of all the predicted systems are linked with other proteins that play different roles during bacterial processes (Fig. 2; Fig. S2), supporting the idea that those systems may have a pivotal function besides the response to stress, as it has been described for other bacteria (18). As previously proposed (52), we need to start thinking about the TA systems not only as independent units but also as part of a complex network, which are abundant in some genomes and can play a myriad of roles besides regulating the bacteria’s response to stress.

### Deletion of a putative HigBA system results in increased expression of redundant systems from the same family

**Fig 3:**
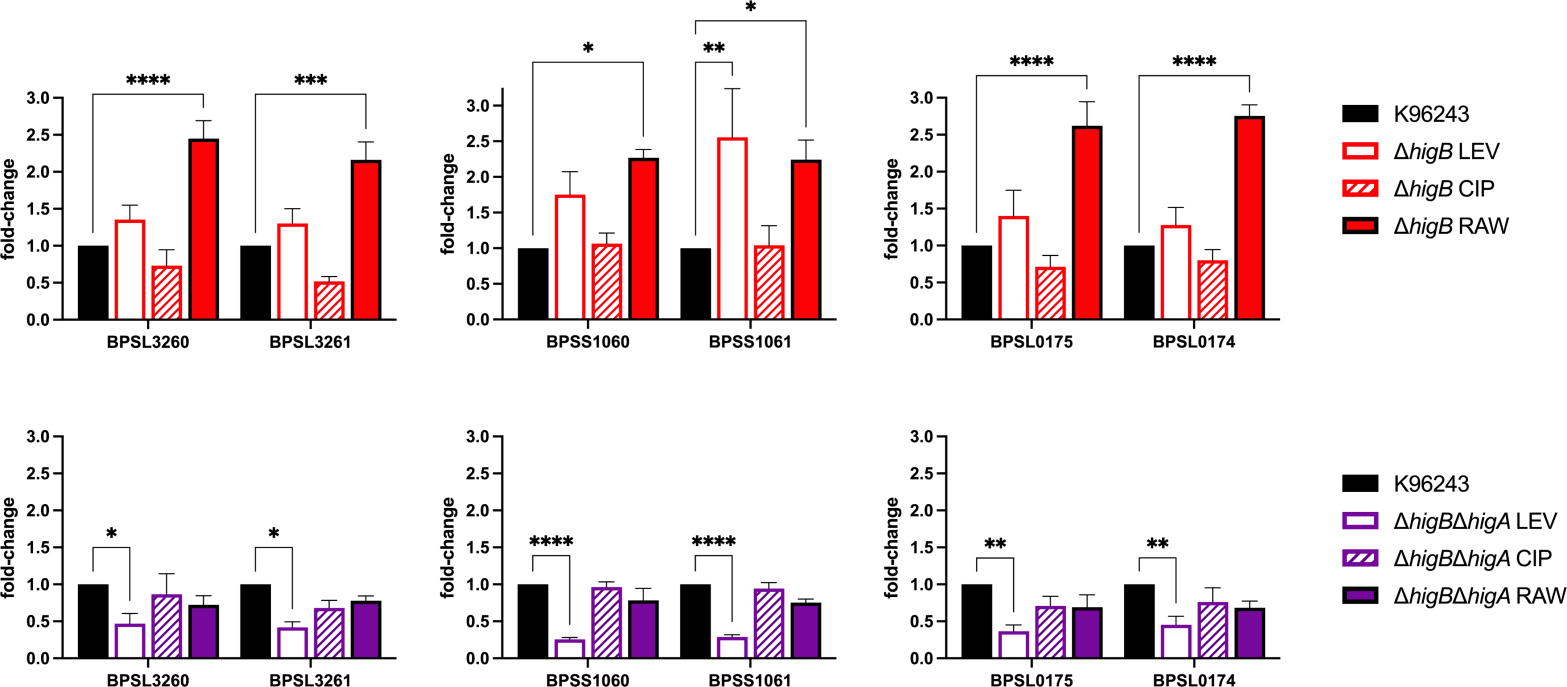
Expression of predicted toxin-antitoxin HigBA systems under stress conditions in ∆BPSL3343 (∆*higB*) or ∆BPS3343-∆BPS_RS18025 (∆*higB*∆*higA*) mutants. Single mutant ∆*higB* or double mutant ∆*higB*∆*higA* strains [ICG001 and ICG002, respectively, from reference (9)] were exposed to the stress conditions that showed differential survival among the mutants and the wild type (LEV: levofloxacin, CIP: ciprofloxacin, RAW: intracellular survival bacteria). The expression of the other three couple of genes predicted as HigB-HigA was evaluated under different conditions, using the expression of the parental strain as control. Two-way ANOVA was performed to establish the significance in each group compared with wild type. Only significant differences are shown: * *P* < 0.05; **P < 0.01; ***P < 0.001; ****P < 0.0001.

Some researchers believe that TA systems are redundant in bacterial genomes (10, 53), and hence the lack of one of those systems is compensated by the expression of the others as backups under specific conditions (9, 22). Because we have previously established the stressful conditions in which BPSL3343-BPS_RS18025 exhibited significantly differential expression patterns (i.e., after antibiotic exposure) and phenotypes in the knockout strains (i.e., levofloxacin exposure) (9), we analyzed the expression of the other three putative HigBA system genes under those conditions, comparing the wild-type strain with the different mutants (Fig. 3). Each toxin-antitoxin pair of genes exhibited the same tendency in all conditions, indicating they may be activated under the same circumstances, either having increased expression once *Bpm* is phagocytosed or during exposure to levofloxacin when the toxin is not present. Interestingly, levofloxacin treatment led to similar patterns of gene repression in both the TA knockout and WT strains, suggesting that another TA system is providing a compensatory role. That could additionally indicate that the antitoxin BPS_RS18025 (HigA) is playing a role over the other systems once its natural counterpart is missing or is causing the other systems to become more activated if the toxin BPSL3343 (HigB) is not acting over its mRNA target. However, more studies will be needed to decipher the interplay of this complex network of these incompletely understood systems (3, 6–9).

### Mutant construction and phenotypic characterization revealed that the deletion of TA genes did not affect normal *in vitro* growth

The *Bpm* K96243 genome encodes at least four putative HigBA systems, suggesting that the protein products of these systems might play a role in various cellular processes. Since the lack of one of the toxins of these TA systems enhances the expression of others under specific conditions, we generated different isogenic mutants to prove the predicted compensatory role as follows: toxin mutant (∆BPSL3261), antitoxin mutant (∆BPSL3260); system mutant (∆BPSL3260 ∆BPSL3261); double toxin mutant (∆BPSL3261 ∆BPSL3343 also named ∆BPSL3261 ∆*higB*), and system mutant together with the lack of the previously described toxin (∆BPSL3260 ∆BPSL3261 ∆*higB*). The single mutants and the system mutant were constructed to evaluate the role of that specific system in previously described conditions associated with the expression of HigBA, while the other constructs were used to decipher a putative crosstalk between the members of that system family.

First, the fitness of all the strains was evaluated in different media to check if the lack of any of those genes alters the normal growth rate of the bacteria (Fig. S3). We observed no differences in any of the mutants’ ability to grow in nutrient-rich medium, minimal medium, or host-like medium. Colony morphology was also evaluated by growing the bacteria on Ashdown agar, but no significant differences in shape, color, or size were found either. Therefore, we can conclude that the differences found among the mutants under the different tested conditions were the result of the stressful conditions and not because of fitness differences among the strains.

### Persister-induction after supra-lethal antibiotic concentrations highlights the stress-dependent nature of the TA systems’ activation

**Fig 4:**
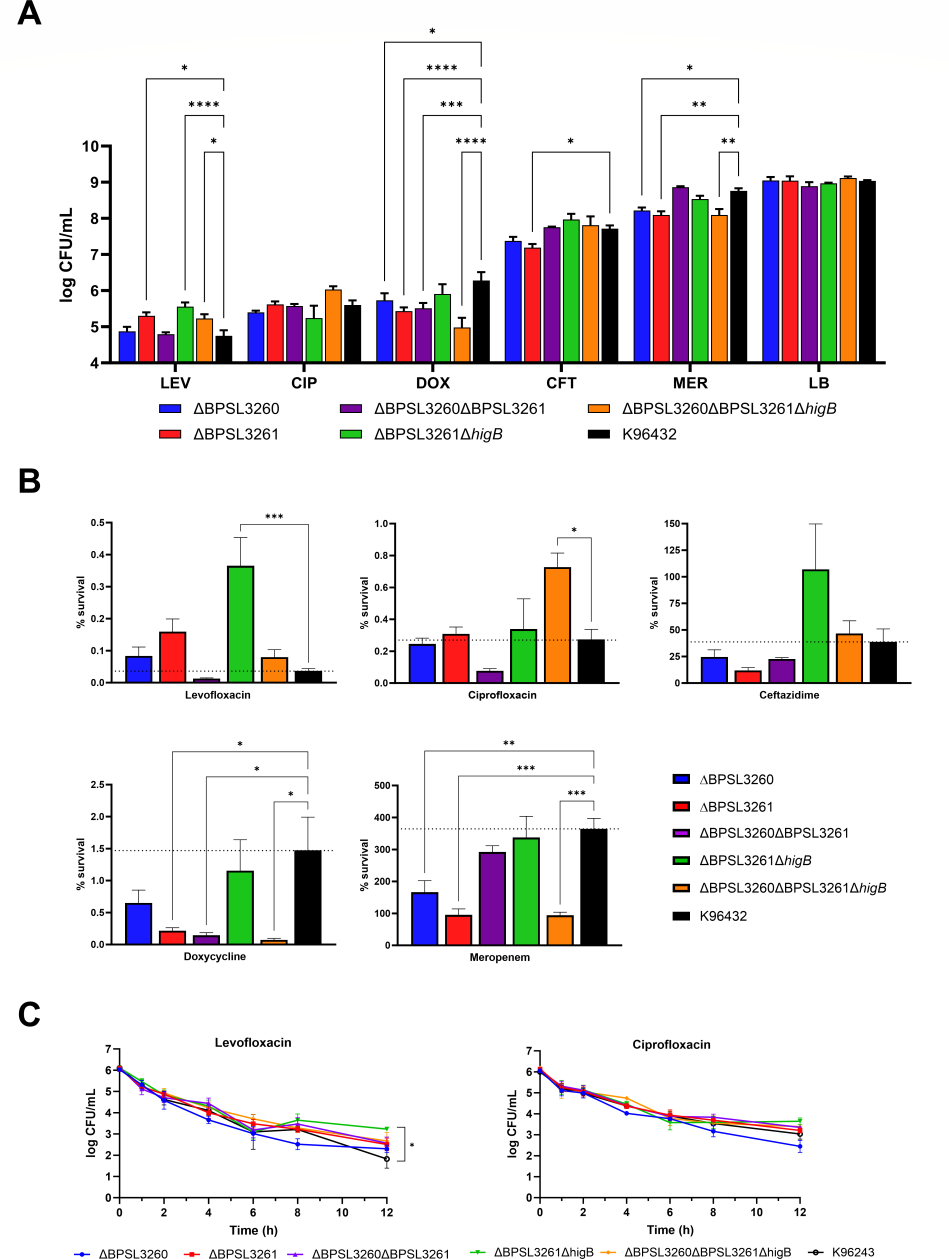
Survival under supra-lethal antimicrobial treatment. (**A**) Total bacterial survival, (**B**) percentage based on input (1 × 10^8^ CFU/mL) after 24 h of antibiotic exposure (considered persisters); (**C**) killing curves in presence of supra-lethal concentrations of fluoroquinolones for 12 h in agitation. Survival increase was observed under levofloxacin treatment for the single or double mutant for the toxins *higB* (∆BPSL3261∆*higB*) (**A**), even showing an accumulative effect in the double mutant under the same conditions (B; upper left graph). Reduced survival was also found in single mutants when bacteria were grown in presence of meropenem. (**C**) Slow killing curves is a persister characteristic, with a sub-population that remains alive besides the bactericidal effect of the antibiotic; after 12 h of treatment, the double mutant ∆BPSL3261∆*higB* also exhibited significantly better survival than its parental strain under levofloxacin treatment. LEV, levofloxacin; CIP, ciprofloxacin; DOX, doxycycline; CFT, ceftazidime; MER, meropenem. Graphs represent the average of three independent experiments in triplicate with standard error (SEM). Dash lines (**B**) indicate the wild type average in each condition. (**A and B**) Two-way ANOVA or (C) multiple *t* test were performed to establish the significance in each group compared with the wild type. Only significant differences are shown: * *P* < 0.05; ** *P* < 0.01; *** *P* < 0.001; **** *P* < 0.0001.

One of the most important and well-described stresses that triggers the persister phenotype through toxin-antitoxin activation is bacterial exposure to antimicrobial compounds. Therefore, we exposed the various TA system mutants to different clinically relevant antibiotics at supra-lethal concentrations to avoid tolerance, as previously described (8, 9, 37). Previously, we proved the role of a putative HigB (BPSL3343) in levofloxacin survival as well as both system components (HigA and HigB) in ciprofloxacin survival (9). Independently of the efficacy of the antibiotics assessed as bactericidal or bacteriostatic compounds, in all the cases, supra-lethal concentrations of each of them resulted in the formation of a subpopulation of bacterial cells that remained alive as a so-called “persister” subpopulation. As shown in Fig. 4A, all the constructs that retained the antitoxin genes but had lost one or both toxin genes (∆BPSL3261; ∆BPSL3261 ∆*higB*; ∆BPSL3260 ∆BPSL3261 ∆*higB*) exhibited increased survival compared with the wild type. When we compared the numbers as percentages (Fig. 4B), we can see how the double toxin mutant ∆BPSL3261 ∆*higB* has a much higher survival rate than the other mutants, suggesting a cumulative effect when both genes are removed. However, when ciprofloxacin is present, the survival trend is quite different. In this case, the system mutant (∆BPSL3260 ∆BPSL3261) shows a clearly reduced survival rate, albeit falling shy of statistical significance (Fig. 4B). Interestingly, the double toxin mutant that exhibited a significantly higher survival in levofloxacin showed no difference in any of the other conditions assessed, while most of the other mutant strains exhibit a reduced survival compared with the wild type, mainly in doxycycline and meropenem. However, no general trend can be concluded from the analysis based on the missing genes, but their absence somehow impacts the bacteria’s ability to survive under high antibiotics’ concentrations. Such complexity has previously been reported in other bacteria (9, 54, 55). What we can conclude is that ∆BPSL3261 only has better survival under levofloxacin treatment; the same survival as the wild type in the presence of ciprofloxacin; and significantly reduced number of persisters when exposed to doxycycline, ceftazidime, and meropenem (Fig. 4A), highlighting once more how these systems’ impact on cellular processes are strictly dependent on the kind of external stress (9, 56). These data are similar to the previously described report for the other HigBA system encoded by BPSL3343 (9), except that the mutant did not exhibit different survival under doxycycline or ceftazidime treatment. This suggests that distinct members of the same TA family can play varying roles in mediating tolerance to different antibiotics. Surprisingly, the antitoxin mutant of that system was not viable, while in the case of BPSL3260-BPSL3261, the lack of the antitoxin did not have any impact on bacterial fitness. The role of TA systems in bacterial persistence is still controversial because there are as many reports that succeed in linking the TA functions with the dormant state as those that do not find any connection (12, 54). According to the data collected from our mutants, this could be explained through different hypotheses: (i) each system responds to specific stimuli to activate the persisters formation; (ii) not all the chromosomal systems may play a role in the switching phenotypes, but some of them do; (iii) the high number of TA systems in prokaryotic genomes ensures the bacterial homeostasis is achieved even when not all of the systems are active.

The phenotypic variants designated as persisters have been traditionally described by slower killing forms in antibiotic survival curves (50, 55). We have above described the differential survival of the isogenic mutants for the system BPSL3260-BPSL3261 under different antibiotic treatment conditions (Fig. 4) and have previously seen a differential survival in the presence of fluoroquinolones in another HigBA system mutant (9). Therefore, we evaluated the killing curves of all the previously described strains in the presence of supra-lethal concentrations of levofloxacin and ciprofloxacin (Fig. 4C), as it is well described that this antibiotic family is a major trigger of the persister state in many bacteria (9, 37, 46, 53, 57). Like the data from survival at 24 h in static conditions (Fig. 4B), the double toxin mutant ∆BPSL3261 ∆*higB* exhibited significantly higher survival in the presence of levofloxacin compared with the parental strain but not in the presence of ciprofloxacin (Fig. 4C). Because the trends seen in these conditions are consistent, we can conclude that the survival observed under those treatments is due to the formation of persisters, in agreement with its definition (55).

### Biofilm formation in the presence of antibiotics exhibited an inverse relationship between bacterial survival and development of biofilms

**Fig 5:**
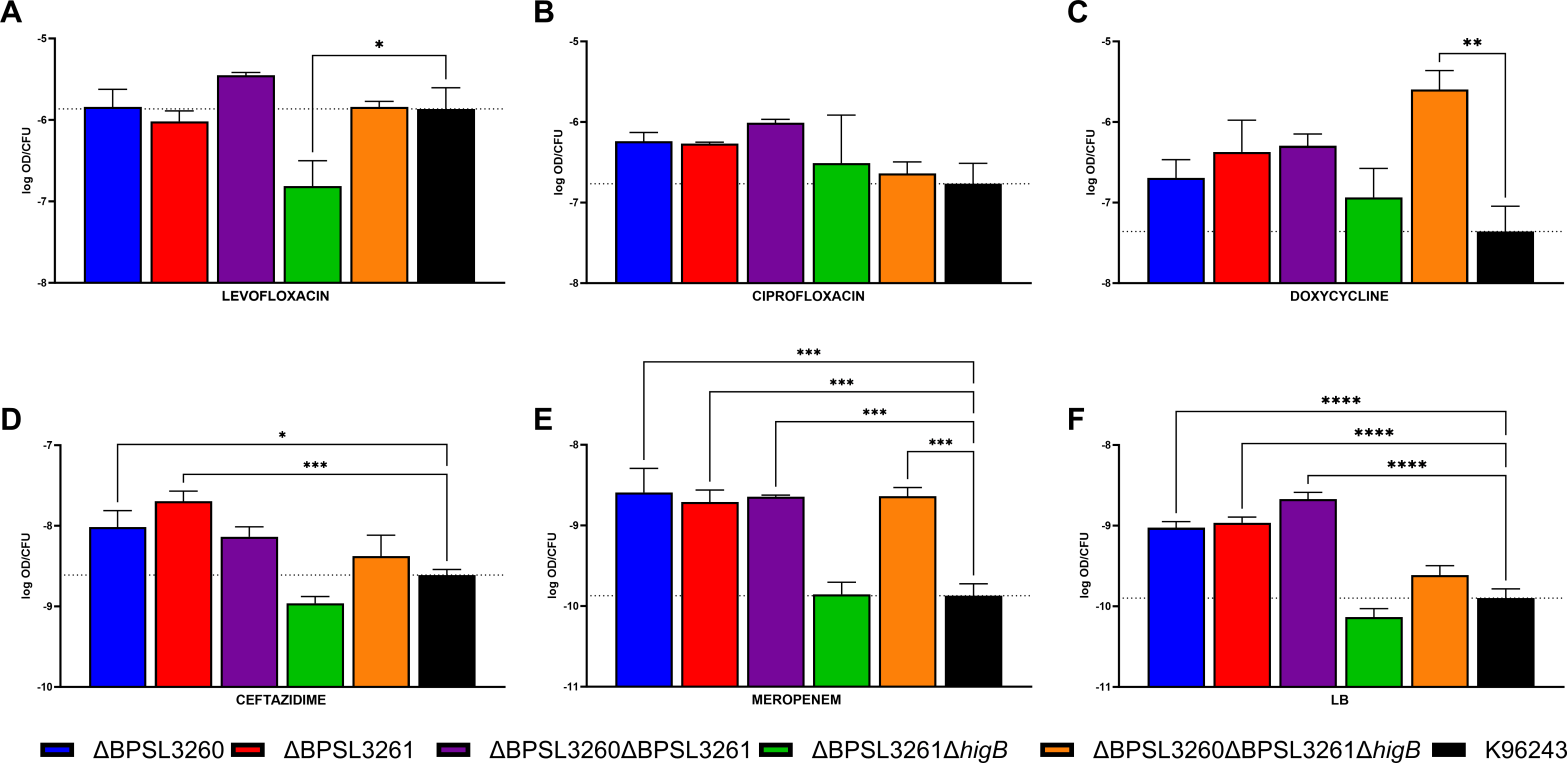
Biofilm formation under supra-lethal antibiotic concentrations. Bacteria were grown statically for 24 h in presence of supra-lethal concentrations of different antibiotics (A, levofloxacin; B, ciprofloxacin; C, doxycycline; D, ceftazidime; E, meropenem) or only LB (**F**); after incubation, survival bacteria were plated, and biofilm stained with crystal violet for absorbance quantification. Because of the differential survival under different conditions and among the strains, the absorbance was normalized with the output bacteria [as previously described (38)]. Bars represent the average of three independent experiments in triplicate +SEM. Two-way ANOVA was performed to establish the significance in each group compared with wild type. Only significant differences are shown: * *P* < 0.05; ** *P* < 0.01; *** *P* < 0.001; **** *P* < 0.0001.

Another contributing factor to the treatment failure in many bacterial infections, including those caused by *Bpm,* is their ability to form biofilms, which increases the possibility of relapse (58). Because so many *Bpm* factors and gene products could be involved in biofilm formation [extensively reviewed in reference (58)], it is not traditionally considered a virulence factor *per se*, although it has been previously linked with higher adherence to epithelial cells *in vitro* and the possibility of disease relapse (59). Therefore, we hypothesize that if the mutants exhibited differential survival upon antibiotic exposure, biofilm formation may also be impaired due to the link between antibiotics and adherence. As such, different mutants were exposed to supra-lethal concentrations of five different clinically relevant antibiotics under static growth conditions and their biofilm formation was assessed and normalized to the number of surviving bacteria (Fig. 5A through E), as previously described (9, 38).

Under levofloxacin exposure conditions, there seems to be an inverse relationship between bacterial survival (Fig. 4B) and biofilm formation upon the same treatment (Fig. 5A). This may indicate that biofilm development and the protection that it confers against the unfavorable environment may be needed more when the bacteria exhibit higher sensitivity to the antibiotic. The same trend is observed in most of the conditions, though the differences are not always significant. For example, there was no difference in biofilm formed under ciprofloxacin exposure, though it appears higher (not significantly) in the TA system mutant ∆BPSL3260 ∆BPSL3261 (Fig. 5B). It also shows a clear decrease in viability compared with the wild type (Fig. 4B). Under doxycycline, ceftazidime, and meropenem exposure, most of the mutants exhibited a reduced survival compared with the wild type under the same conditions (Fig. 4B) and their adherence capacity was enhanced. Also, in meropenem’s case, the double toxin mutant ∆BPSL3261 ∆*higB* had the same survival as the wild type, and it also exhibited the same amount of biofilm (Fig. 5E). Interestingly, the single mutants (∆BPS3260 and ∆BPSL3261) and the double mutant (∆BPSL3260 ∆BPSL3261) also exhibited a higher biofilm formation ability compared with the wild type in non-supplemented LB media, while they did not exhibit growth differences. This phenomenon was also observed in ∆*higB* ∆*higA* from our previous work, but not in ∆*higB* (9). Although there is not a total correlation in all the tested conditions for all the strains, and considering the lack of knowledge about how those systems impact the bacteria viability (13), there is a clear trend that allows establishing a generalized inverse relationship between the bacterial formation of persisters in response to antimicrobial treatment and its ability to form biofilm under the same antibiotic exposure.

### Overexpression of plasmid-encoded HigBA in *E. coli* did not result in growth arrest

Traditionally, canonical toxin-antitoxin systems have been characterized *in vitro* through growth arrest in inducible systems. However, more recent data suggest that not all systems may impact growth under the same conditions or be functional in all host species. For that reason, we evaluated the impact of the BPSL3260-BPSL3261 system in an inducible vector using *E. coli* DH10 as the carrier strain (8, 9). We then compared the growth of the empty vector and the vector carrying the antitoxin (BPSL3260), the toxin (BPSL3261), and the entire system (BPSL3260-BPSL3261) in induced versus non-induced *E. coli* (Fig. S4A) as well as in LB or M9 induced once for longer timepoints (Fig. S4B). However, there were no differences among any construct under any of the tested conditions. This may indicate that neither of the system components is toxic under those growing conditions, or that its target is not present in the recipient strain, suggesting that the toxin functions in a strain- or species-dependent manner, or changes are too subtle to observe under growth conditions (60).

The traditional way to describe a bona fide toxin is to demonstrate growth arrest induced by ectopic expression; however, an increasing number of authors believe that this is not the only way to define a TA system. For example, many of them are proven to be expressed under normal growing conditions, which suggests their role in different pathways and that it is not necessarily required to be a “true” toxin (61, 62). It has also been proven for *Bpm* and other bacteria that ectopic expression of the toxins does not always cause growth arrest, with differences even from the same family of toxins or in different hosts (7, 60, 63).

### Toxin-antitoxin system mutant strains exhibit impaired intracellular survival and persistence that do not correlate with *in vitro* virulence

The intracellular life cycle of *Bpm* is well known, as well as its ability to survive and replicate inside macrophages (64). However, the molecular mechanisms that allow this process are not well understood. Because this phenomenon is also known to be linked with persistence, we evaluated the ability of the different mutants to be taken up by macrophages and once engulfed, their ability to survive intracellularly. Additionally, cells were also treated with the cell-permeable antibiotic levofloxacin since we have already found that this treatment has a differential impact on the mutants.

**Fig 6:**
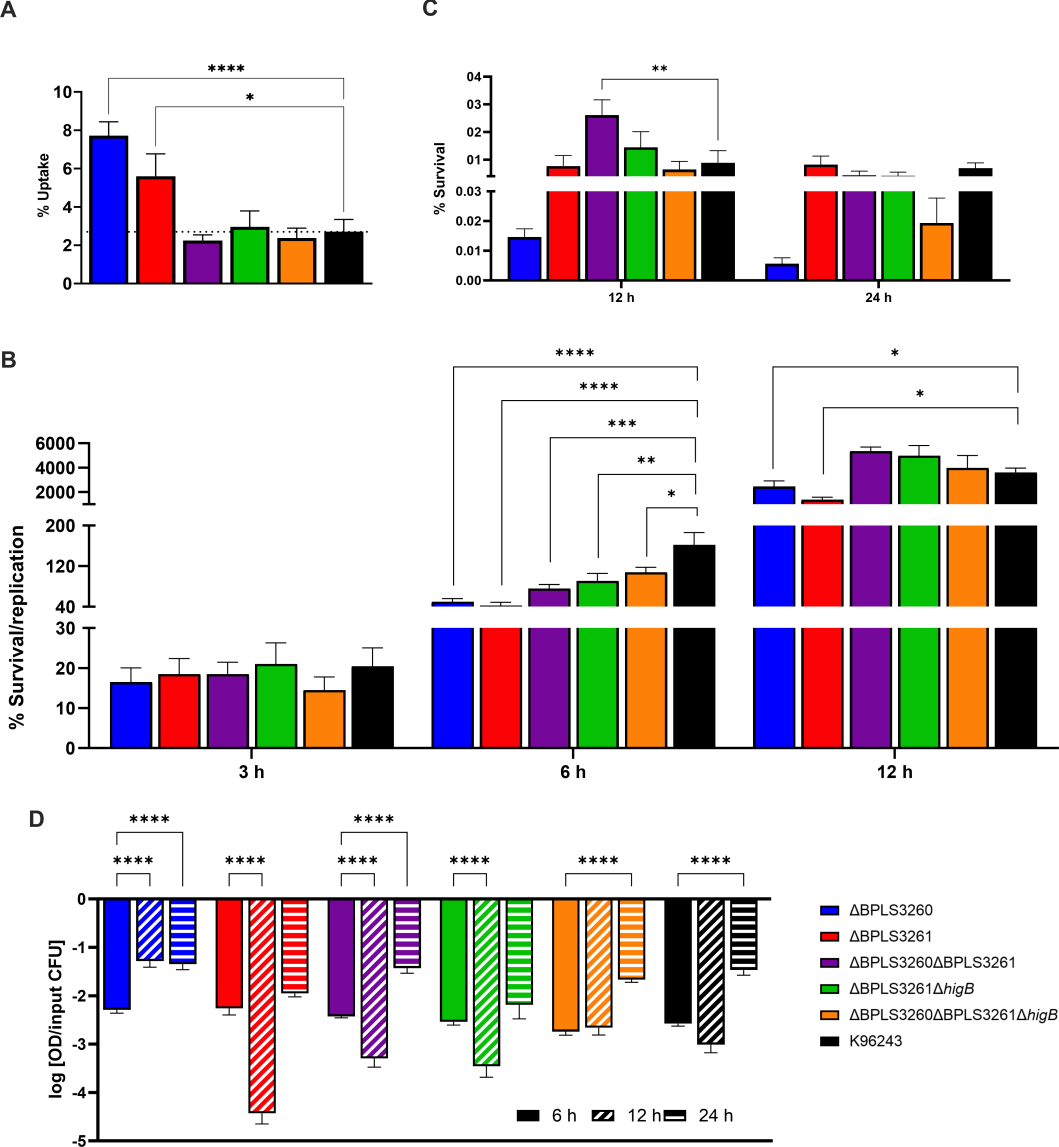
Uptake, intracellular survival, and biofilm formation ability under macrophage-induced-persister conditions. Murine RAW macrophages were infected with the different strains at MOI 10 for 30 min. (**A**) Uptake percentage was calculated based on the input bacteria. After 30 min of the invasion phase, media were removed and replaced with fresh DMEM media supplemented with kanamycin (**B**) or levofloxacin (**C**). Bars represent the average of two independent experiments in quadruple +SEM. (**D**) Macrophages were infected as previously described, and the recovered bacteria after 6 h (treated with kanamycin) and 12 or 24 h (treated with levofloxacin) were used to inoculate fresh LB and evaluate their biofilm formation ability. Colored bars represent the biofilm from bacteria recovered after 6 h of kanamycin-treated macrophages (considered control for statistics), stripped bars represent the biofilm formed by bacteria recovered from macrophages treated with levofloxacin for 12 h (diagonal stripped) or 24 h (horizontal stripped). Bars represent the average of three independent experiments in triplicate +SEM. One-way (**A**) or two-way ANOVA (**B–D**) tests were performed to establish the significance in each group compared with wild type. Only significant differences are shown: * *P* < 0.05; ** *P* < 0.01; *** *P* < 0.001; **** *P* < 0.0001.

Phagocytosis was evaluated in the murine macrophage cell line RAW 264.7 after 30 min of infection at MOI 10. Both single mutants exhibited an enhanced uptake (Fig. 6A) compared with the WT, suggesting that these bacteria are engulfed faster by macrophages than the other strains. The intracellular survival of the different strains was assessed at 3, 6, and 12 h post-infection (Fig. 6B). No difference in survival after 3 h infection was seen, though all the mutants showed an impaired survival at distinct levels after 6 h. While the survival percentage for single mutants is between 40% and 50%, it ranges from 75% to over 100% for the other mutant strains. At 12 h, the bacteria is replicating inside the cells and some infected cells are beginning to die off (65). Thus, the 12-h time point better depicts the ability of the mutants to replicate within and subsequently kill the host cell. At this time point, we only found significant differences in the single mutants, both of which exhibited reduced bacterial counts compared with the WT strain (Fig. 6B). This may suggest that lack of the system counterpart (either the toxin or the antitoxin) can have a higher impact on the intracellular life cycle than the lack of the entire system.

Taking together the intracellular survival defect (Fig. 6B) with the different survival seen under levofloxacin treatment (Fig. 4B), we combined these two assays, using the cell-permeable antibiotic levofloxacin after phagocytosis, to see the effect of it over the different mutants, as previously described (9). Although there was no statistical significance, a clear defect in survival in the antitoxin-lacking strain was seen at both time points (Fig. 6C), while no effect on the double toxin mutant survival was observed, highlighting once more how the effect of these systems is dependent on the specific stimulus and environmental conditions.

Biofilm formation is sometimes linked with the persister phenotype (17, 37, 62, 66) and we have already established differences among strains under diverse stressful conditions (supra-lethal antimicrobials exposure) (Fig. 5). Therefore, we evaluated biofilm formation using intracellular bacteria recovered from macrophages at different time points. Our goal was to evaluate if the inverse relationship between the survival and ability to form biofilms observed under antibiotic treatment was shared by the intracellular bacteria (Fig. 5). For that purpose (9), we used the phagocytosed bacteria to inoculate fresh LB broth and incubate it statically for 24 h. The biofilm was quantified and normalized with the input CFU to eliminate different survival rates among the strains and the time points (Fig. 6D). We also evaluated the effect of levofloxacin treatment in this condition, comparing the biofilm formed by bacteria collected after 6 h of macrophages infection when the bacteria are not highly replicating yet (Fig. 6B), with those at 12 and 24 h of macrophage infection when the cells were subjected to levofloxacin treatment (Fig. 6C). The 6 h infection data were used for comparison since there were no differences at this time point among the normalized biofilm value of the strains. Each strain exhibited different behavior, but some trends were found. The antitoxin mutant ∆BPSL3260 exhibited lower survival when macrophages were subjected to levofloxacin treatment (Fig. 6C), with similar values between both time points, and it showed a higher biofilm formation ability at both timepoints. Interestingly, both single (∆BPSL3261) and double toxin mutant strains (∆BPSL3261 ∆*higB*) exhibited the same pattern, with reduced biofilm in samples from 12 h but the same after 24 h, which is the opposite of what happened in the triple mutant (∆BPSL3260 ∆BPSL3261 ∆*higB*) and the wild type (Fig. 6D). However, the system mutant ∆BPSL3260 ∆BPSL3261 showed both behaviors, with reduced biofilm in bacteria from 12 h infection and increased from survivors from 24 h, which inversely correlates with its survival (Fig. 6C), which was also found for biofilm formed under supra-lethal antimicrobial conditions (Fig. 5). Considering the high variability of those systems and their behavior under different stress combinations, we can conclude, however, that there is a general common trend between the bacteria’s ability to survive and its capacity to form biofilm after being exposed to unfavorable conditions, independently of the nature of those conditions (67).

### *In vivo* bacterial infection model displayed no difference in virulence but showed bacterial preference for organ colonization after levofloxacin treatment

**Fig 7:**
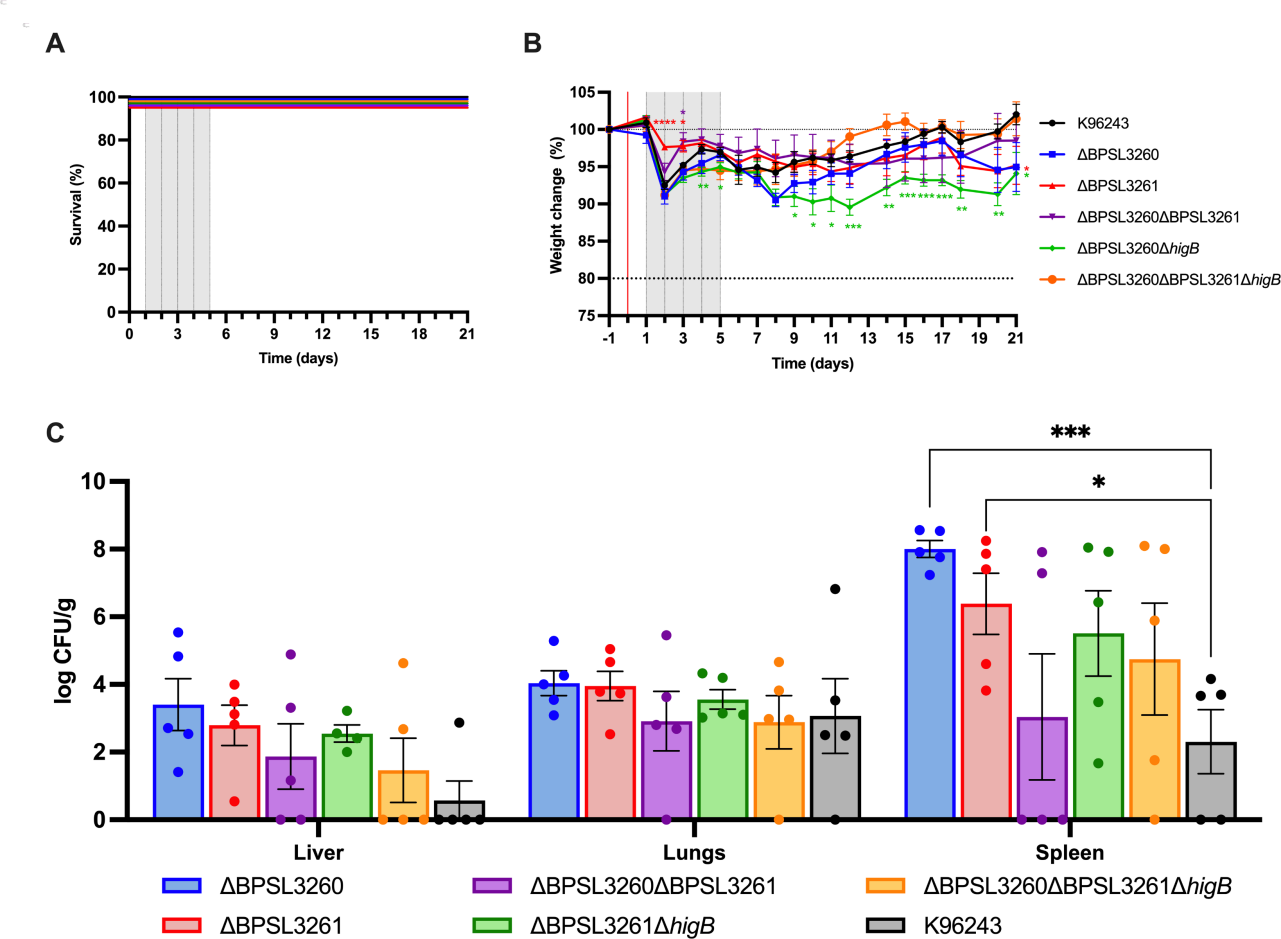
Persistence of different *B. pseudomallei* strains *in vivo* after levofloxacin treatment. (**A**) Survival and (B) weight change during the 21 days of infection. (**A and B**) Grey shadow represents the days of antibiotic treatment. In (B) panel, red vertical line represents the challenge day and horizontal dotted line shows the humane endpoint established (80% of starting weight). (**C**) Bacterial burden in lungs, liver, and spleen of *n* = 5 mice challenge with each strain ±SEM. Multiple *t* test (**B**) or two-way ANOVA (**C**) were used to establish statistical differences using wild-type data as control. Only significant differences are shown: * *P* < 0.05; ** *P* < 0.01; *** *P* < 0.001.

After evaluating the different mutant strains’ behavior through different *in vitro* assays, we decided to examine their role in an animal model. The ability of *Bpm* to infect and cause lethal disease in mice is well known, mimicking some of the human melioidosis symptoms, and it can cause both acute to chronic (latent) infections. Because of the previously described role of this TA system in persistence *in vitro*, we induced a chronic-persister melioidosis infection in a murine model using female BALB/c mice (8, 9). Animals were intranasally infected with the parental strain and the different mutants, and treated for 5 days post-infection with levofloxacin, which pushes the infection into a chronic-persistent state, that mimics a relapse in humans (3, 10). After that, animals were checked daily for up to 21 days and their weight was recorded. Following euthanasia, organs were collected for evaluation of the bacterial burden (Fig. 7). There were no differences in mortality among the strains (Fig. 7A) since all the animals survived until the established endpoint. No animal exhibited weight loss that would be considered a humane endpoint requirement, but the double toxin mutant ∆BPSL3261 ∆*higB* strain caused significantly higher weight loss compared with the WT strain after the levofloxacin treatment (Fig. 7B). Also, at day 21 post-infection, the mice infected with the toxin mutant ∆BPSL3261 exhibited a significantly lower weight than the control animals (infected with parental strain K96243), which were already gaining weight at that point (Fig. 7B). If we consider this data together with that collected from the *in vitro* survival (Fig. 4A and C), we may hypothesize that those strains that exhibited lower killing activity from levofloxacin *in vitro* also maintain higher survival *in vivo* when they are exposed to the same antibiotic. Due to the systemic dissemination of the pathogen, the bacterial burden was evaluated through CFU enumeration from liver, lungs, and spleen of the infected animals (Fig. 7C) (8, 9). No significant differences among strains were observed in livers or lungs. However, most of the groups exhibited a higher trend of spleen colonization compared with the WT strain, while only the single mutants ∆BPSL3260 and ∆BPSL3261 showed significant differences due to the more consistent values of all the animals in each group (Fig. 7C). These data correlate with that previously collected for the putative HigBA system encoded by BPSL3343-BPS_RS18025, in which the toxin mutant only showed higher burden in spleen (9), while, as previously explained, we could not evaluate the antitoxin mutant alone in that case. Taking all data together, it seems to indicate that the mutants have a preference to become persisters in the spleen, which highlights the importance of understanding the latent infections to fully treat and clear this pathogen. More research is needed to further elucidate the role of these systems in the chronic infections of melioidosis, but all the data clearly indicates a relationship between the system encoded by BPSL3260-BPSL3261, levofloxacin treatment, and persistence.

### Transcriptomic analysis revealed both shared and individual mechanisms in different TA systems

With all the collected data and the several conditions in which the mutants were evaluated, the mechanism behind the preliminary established phenotype was not elucidated. The complexity of concluding a clear phenotype based on our cumulative data is challenging because, as we have previously stipulated, there is no consensus about the role of these systems in persistence, which is further complicated by the lack of information about this pathogen. Nevertheless, we further decided to analyze the expression pattern of the strains, especially in the conditions that showed a clearer and more consistent phenotype. Transcriptomic approaches are gaining importance among researchers to narrow the pathways involved under different conditions, especially when the available information is scarce or null (16, 17, 54, 68, 69).

**Fig 8:**
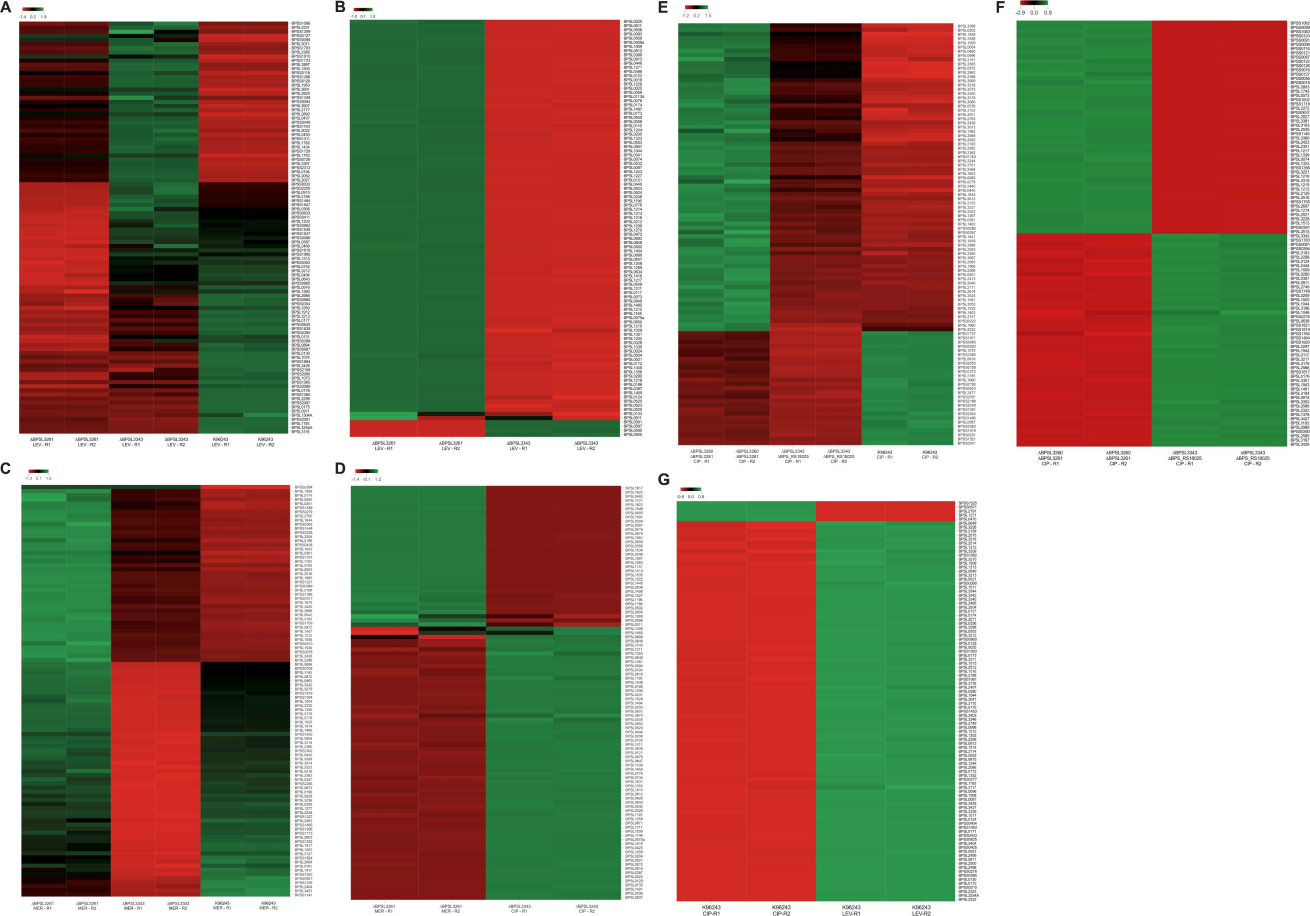
Heatmaps generated from RNA-seq data. Toxin mutants and systems mutants that exhibited similar behaviors under specific antibiotic treatments (Fig. **S5**) were submitted for RNA-sequencing in duplicate, and differential comparisons were established. (**A and B**) Toxin mutants and wild type subjected to levofloxacin treatment, comparing the shared pattern between mutants against wild type (**A**) or the difference between both mutants (**B**). (**C and D**) Toxin mutants and wild type subjected to meropenem treatment, comparing the shared pattern between mutants against wild type (**C**) or the difference between both mutants (**D**). (**E and F**) System mutants (both toxin and antitoxin deleted) and wild type subjected to ciprofloxacin treatment, comparing the shared pattern between mutants against wild type (**E**) or the difference between both mutants (**F**). **G**) Differential expression pattern in wild-type K96243 when subjected to levofloxacin versus ciprofloxacin treatments. Each panel shows the top 100 genes that are most significantly differentiated and expressed in each condition. Genes nomenclature was obtained for all the panels from www.burkholderia.com database (last accessed December 2023) for the reference strain K96243.

Thus, we compared the results obtained from the mutants generated in this work and the previous one (9) and we found common survival patterns between the levofloxacin, ciprofloxacin, and meropenem treatments (Fig. S5). Briefly, both toxin mutants (∆BPSL3261 and ∆BPSL3343 -*∆higB*-) exhibited an increased survival under levofloxacin treatment that was not present in the TA system or the antitoxin mutants, while those same mutants shared a reduced survival under meropenem exposure; however, the lack of both systems (∆BPSL3260 ∆BPSL3261 and ∆BPSL3343 ∆BPS_RS18025 -∆*higB* ∆*higA*) diminished the bacteria’s ability to survive in presence of lethal concentrations of ciprofloxacin. Therefore, we prioritized those conditions to analyze their differential expressions through RNA-seq. All the comparison combinations were manually established using the RaNA-seq platform (41). Figure 8 shows the heatmaps obtained after all the established combinations (i.e., Fig. 8A, both toxin mutants compared with the WT under levofloxacin treatment; Fig. 8B, comparison between toxin mutants under levofloxacin treatment; Fig. 8C, both toxin mutants compared with the WT under meropenem treatment; Fig. 8D, comparison between toxin mutants under meropenem treatment; Fig. 8E, both system mutants compared with the WT under ciprofloxacin treatment; Fig. 8F, comparison between system mutants under ciprofloxacin treatment; Fig. 8G, comparison between the ciprofloxacin and levofloxacin treatment in WT). Only the top 100 most significant genes are shown in each case, and the list of them with the shared expression among conditions is listed in Table S1. In Fig. 8, the genes nomenclature was assigned based on the listed genes in the *Burkholderia* Genome Database for the reference strain K96243.

Our objective was to identify the genes that show the same expression pattern in mutants when exposed to the same stressful conditions compared with WT (Fig. 8A, C and E), and the genes that are differentially expressed between mutants that exhibit similar phenotype under the same conditions (Fig. 8B, D and F). Thus, when the toxin mutants were exposed to levofloxacin (Fig. 8A), the sequencing revealed that they shared only a few overexpressed genes that are repressed in WT, among them is BPSS0099, which encodes for a Type 6 Secretion System (T6SS) component (*tssD*). However, the mutants shared some relevant commonly repressed genes that were overexpressed in WT: BPSL3213, which encodes for a ribosomal protein (*rplC*); BPSL3426, two-component regulator; and the type II toxins BPSS0390 (*hicA*), BPSS1060 (*higB*), and BPSL0175 (*higB*). Only a few genes are repressed in ΔBPSL3261 compared with ΔBPSL3343, and all of them are identified as hypothetical proteins (Fig. 8B). However, BPSL0591-BPSL0590 shared homology with the putative TcdB and TccC toxins (70). Nevertheless, there are many genes significantly overexpressed in ΔBPSL3261 but repressed in ΔBPSL3343, like the BPSL1405 that encodes for the Lon protease, the operon related with NADH pathway (BPSL1211-BPSL1218), the antitoxin BPSL0174 (*higA*), the toxin BPSL0559 (*hipA*), as well as several transcriptional regulators (BPSL0059, BPSL0647, BPSL1269, BPSL0117, BPSL1495, and BPSL1165).

Those two toxin mutant strains (ΔBPSL3261 and ΔBPSL3343) also showed similarly reduced survival when exposed to meropenem (Fig. S5), and interestingly they only shared a gene significantly overexpressed in both strains but repressed in WT under those conditions—BPSS0394, which encodes for the antitoxin BrnA (Fig. 8C). Surprisingly, the BPSL0174 (antitoxin HigA) appears as the top overexpressed gene in ΔBPSL3261 while repressed in the other two strains. Among the shared repressed genes, only BPSS1141 has a predicted function as a transcriptional regulator. To better understand how the lack of those genes impacts the bacteria, we then compared the differential expression of genes between both toxin mutants under meropenem exposure (Fig. 8D). The genes overexpressed in ΔBPSL3261 and repressed in ΔBPSL3343 are mostly identified as hypothetical proteins, but two of them encode for transcriptional regulators—BPSL1188 and BPSL1269. On the other hand, among the repressed genes in ΔBPSL3261, many genes that encode for proteins related to ribosomal functions (BPSL1458, BPSL1460, BPSL1461, BPSL1491, BPSL0915, BPSL0916, BPSL0871, BPSL1355, BPSL1206, BPSL1511, BPSL0520, and BPSL0075a) were identified.

When the different strains were exposed to ciprofloxacin, the double mutants lacking the whole TA system exhibited reduced survival compared with the WT (Fig. S5). Consequently, we compared the expression of those three strains subjected to supra-lethal concentration of that antibiotic. Among the most significant ones, there were no shared overexpressed genes between both mutants in contrast with WT, but there were some commonly repressed, among which are some virulence-related genes that encode for T6SS (BPSS0524, *tagE*; BPSS1495, *virA*) and T3SS (BPSS1521, *bprD*; BPSS1391, *bpspB*) components (Fig. 8E). Due to the lack of the antitoxin in both mutants, and since it was previously described as a transcriptional regulator in *Pseudomonas aeruginosa* (16), we can hypothesize that HigA may play a similar role in *Bpm* under the ciprofloxacin treatment. When analyzing the expression between both mutants, among the overexpressed genes in ΔBPSL3260 ΔBPSL3261, it is worthy to highlight the T6SS components (BPSS0098 and BPSS0099), both *gyrA* and *gyrB* (BPSL2521 and BPSL0077), and BPSS0391 (antitoxin HicB) (Fig. 8F). For the other strain (ΔBPSL3343 ΔBPS_RS18025), among the genes significantly repressed in ΔBPSL3260 ΔBPSL3261, we found BPSS0394 (antitoxin BrnA) and BPSS0390 (toxin HicA), and different genes that encodes for ribosomal-related proteins (BPSL0915, BPSL1461, BPSL1942, BPSL1943, BPSL2444, BPSL3194, BPSL3196, BPSL3197, BPSL3209, and BPSL3217). It is important to highlight a putative new TA system, encoded by BPSS1821-BPSS1820, that exhibits identity homology with the MbcTA system from *M. tuberculosis* (71). This is noteworthy since the activation of the toxin has shown a lethal impact on *M. tuberculosis* viability *in vitro* and *in vivo*, which could be used for clinical treatment.

As previously seen (Fig. 4), *Bpm* has a different response and hence persister formation rates once it is subjected to supra-lethal concentrations of different antimicrobials from the same family, such as fluoroquinolones. Thus, we compared the most significant differentially expressed genes in wild-type K96243 once the bacteria were exposed for 24 h to levofloxacin or ciprofloxacin. Only 5 out of 100 most significant differentially expressed genes between these two conditions are overexpressed after ciprofloxacin and repressed under levofloxacin treatments, whereas all the rest exhibited the opposite pattern (Fig. 8G). Among those five repressed genes after levofloxacin exposure, BPSS1525, which encodes for a T3SS component (*bopE*), was found. On the other hand, within the genes significantly overexpressed under levofloxacin treatment, it is worthy to highlight BPSS0390 (toxin HicA), BPSL0174 and BPSS1061 (antitoxins HigA), and BPSS0394 (antitoxin BrnA). Regarding the remaining genes that were significantly overexpressed in levofloxacin but repressed in ciprofloxacin treatment, some transcriptional and response regulators (BPSL3426-3427, BPSL3115, and BPSL0117), as well as genes that encode for ribosomal-related proteins (BPSL2159, BPSL2515, BPSL3318, BPSL3209, BPSL3210, BPSL3213, BPSL3204, BPSL3212, BPSL3211, and BPSL1508) were found.

As it is shown in Table S1, some genes are differentially expressed under different conditions, and some are present in nearly all conditions. Among the most repeated genes, we can highlight: BPSL0911, which encodes for a transmembrane fatty acid desaturase, and was the gene that exhibited differential expression in most conditions and can be related to antimicrobial resistance, persistence, and/or virulence (72); BPSL0124, M48 family peptidase; BPSL1193, hypothetical membrane protein; BPSL1212 (*nuoB*), encoding for NADH dehydrogenase subunit B, also linked with virulence and fitness (73); BPSL0915, *rpmG*, 50S ribosomal protein L33, linked with stress response (74); BPSL1323, heat shock protein (only when comparing mutants between them); and BPSS0394, antitoxin BrnA, whose counterpart was previously linked with persistence in *Bpm* (7, 37). This comparison may pave the way to define the complex network and the systemic impact that those TA system components have under stress conditions.

Finally, since one of the main goals of this work was to unveil the TA systems network, we manually trimmed and represented only the expression of the predicted TA system genes previously listed (9), as well as the new predicted BPSS1820-BPSS1821 (Fig. S6). As we previously hypothesized, the expression of those genes is highly dependent on the stressful conditions the bacteria are subjected to. Although there are some common expression patterns among mutants (Fig. S6A through C), especially those genes that encode for toxins from the same family (i.e., BPSL0175 and BPS1060), each system has a different impact on the other systems, as it was predicted by *in silico* methods (Fig. 2; Fig. S2) and also proved through the whole transcriptomic analysis (Fig. 8).

### Concluding remarks

Understanding the molecular mechanisms that underly melioidosis chronic infections are imperative due to their association with relapse and treatment failure. In this work, we extensively evaluated the role of one of the dozens of toxin-antitoxin systems that this bacterium carries on its genome, under different *in vitro* and *in vivo* conditions linked to bacterial persistence. Not surprisingly, we found high variability among the strains and conditions evaluated. However, some clear trends seem to link the HigBA systems with the bacterial survival under specific treatments. Despite our efforts, including RNA-seq data, the mechanism behind that connection could not be elucidated, though there is no doubt about the need for some of those systems during bacterial survival under stressful conditions. More studies and different approaches will be needed to understand the complex network of TA systems and to fully elucidate their role in bacterial survival.

## Data Availability

The RNA-seq data have been deposited at Sequence Read Archive (SRA) under the accession number PRJNA1113347 (BioProject ID).

## SUPPLEMENTAL MATERIAL

Supplemental material spectrum.00748-24-s0001.pdf Table S1; Fig. S1 to S6.

ASM does not own the copyrights to Supplemental Material that may be linked to, or accessed through, an article. The authors have granted ASM a non-exclusive, world-wide license to publish the Supplemental Material files. Please contact the corresponding author directly for reuse.
