# Supplementary material for: Unraveling the role of toxin-antitoxin systems in Burkholderia pseudomallei: exploring bacterial pathogenesis and interactions within the HigBA families
Source: Microbiol Spectr. 2024 Jun 25;12(8):e00748-24. doi: 10.1128/spectrum.00748-24 (PMC11302019; doi:10.1128/spectrum.00748-24)
Supplement: Supplemental material — Table S1; Fig. S1 to S6. [file spectrum.00748-24-s0001.pdf]

Unraveling the Role of Toxin-Antitoxin Systems in *Burkholderia pseudomallei*: Exploring Bacterial Pathogenesis and Interactions within the HigBA Families.

Itziar Chapartegui-González [a,b], Jacob L. Stockton [a], Sarah Bowser [a], Alexander J. Badten [a,c], Alfredo G. Torres [a,d*]

[a] Department of Microbiology and Immunology, University of Texas Medical Branch, Galveston, TX 77550

[b] Division of Infectious Diseases, ANA Futura Laboratory, Department of Medicine Huddinge, Karolinska Institutet, Stockholm, Sweden 141 52

[c] Institute for Translational Sciences, Galveston, TX 77550

[d] Department of Pathology, University of Texas Medical Branch, Galveston, TX 77550

SUPPLEMENTAL

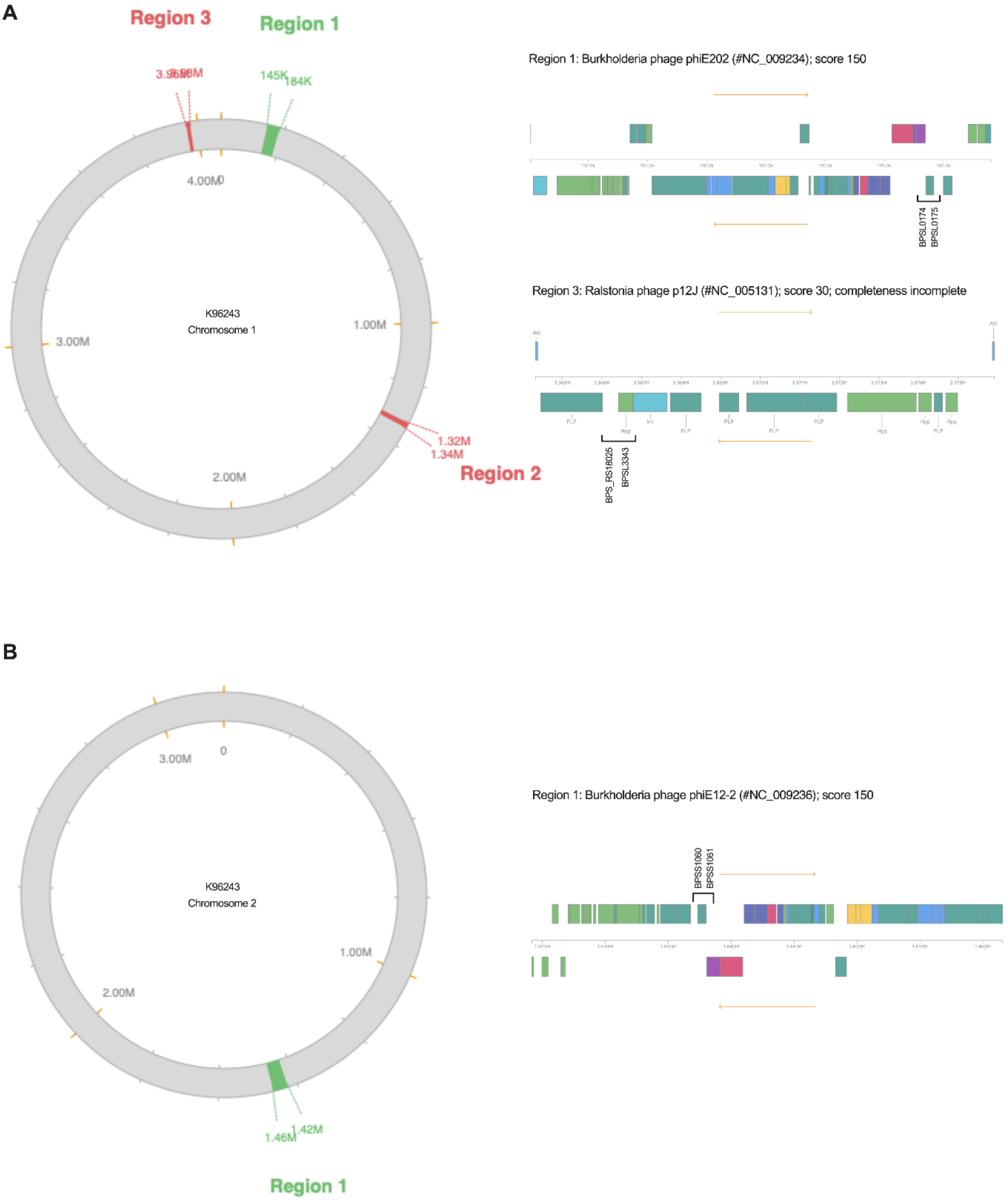

Figure S1. Phages prediction in *B. pseudomallei* K96243 genome through PHASTER. Phages detected in **A)** chromosome 1 or **B)** chromosome 2, and the presence of putative HigBA system within them (manually highlighted using the previously indicated nomenclature).

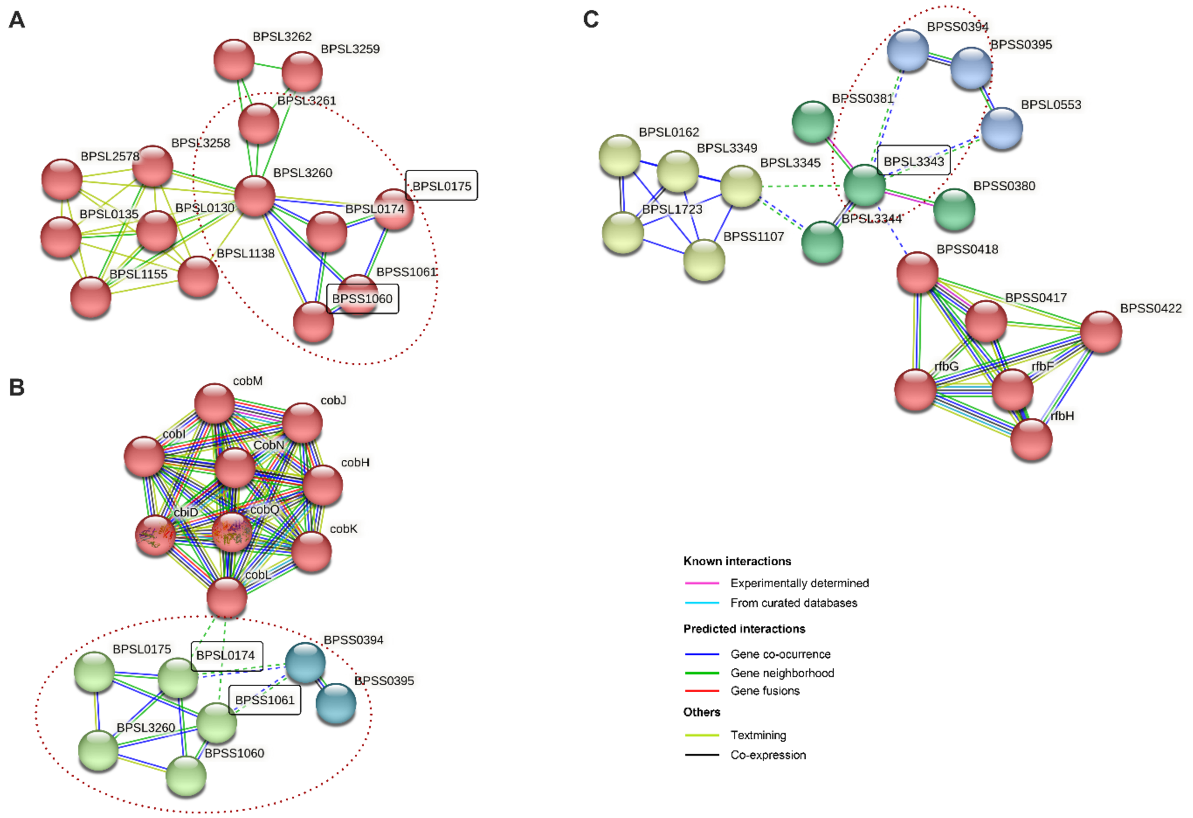

Figure S2. STRING network of the other three predicted system HigBA encoded in *B. pseudomallei* K96243. Networks predicted by the **A)** toxins BPSL0175 and BPSS1060, and the **B)** antitoxins BPSL0174 and BPSS1061, are the same between them, highlighting the fact that the same system was duplicated within the genome. **A)** The toxins are linked with the HigBA system encoded by BPSL3261-BPSL3260 and the connections are made through the antitoxin BPSL3260 (Fig. 2B). **B)** The antitoxins relate to the precorrin synthesis pathway (red cluster), and with the predicted type II TA system BrnT-BrnA, encoded by BPSS0394-BPSS0395 (blue cluster). **C)** The toxin BPSL3343 is the connector among the LPS biosynthesis and transport membrane (BPSS0418) pathways (red cluster), the type II BrnT-BrnA and DNA binding protein (BPSL0553) (blue cluster); DNA-binding regulatory proteins (green cluster); and phage and membrane proteins (yellow cluster). Red dashed lines circle the TA systems components; black squares indicate the origin of the network.

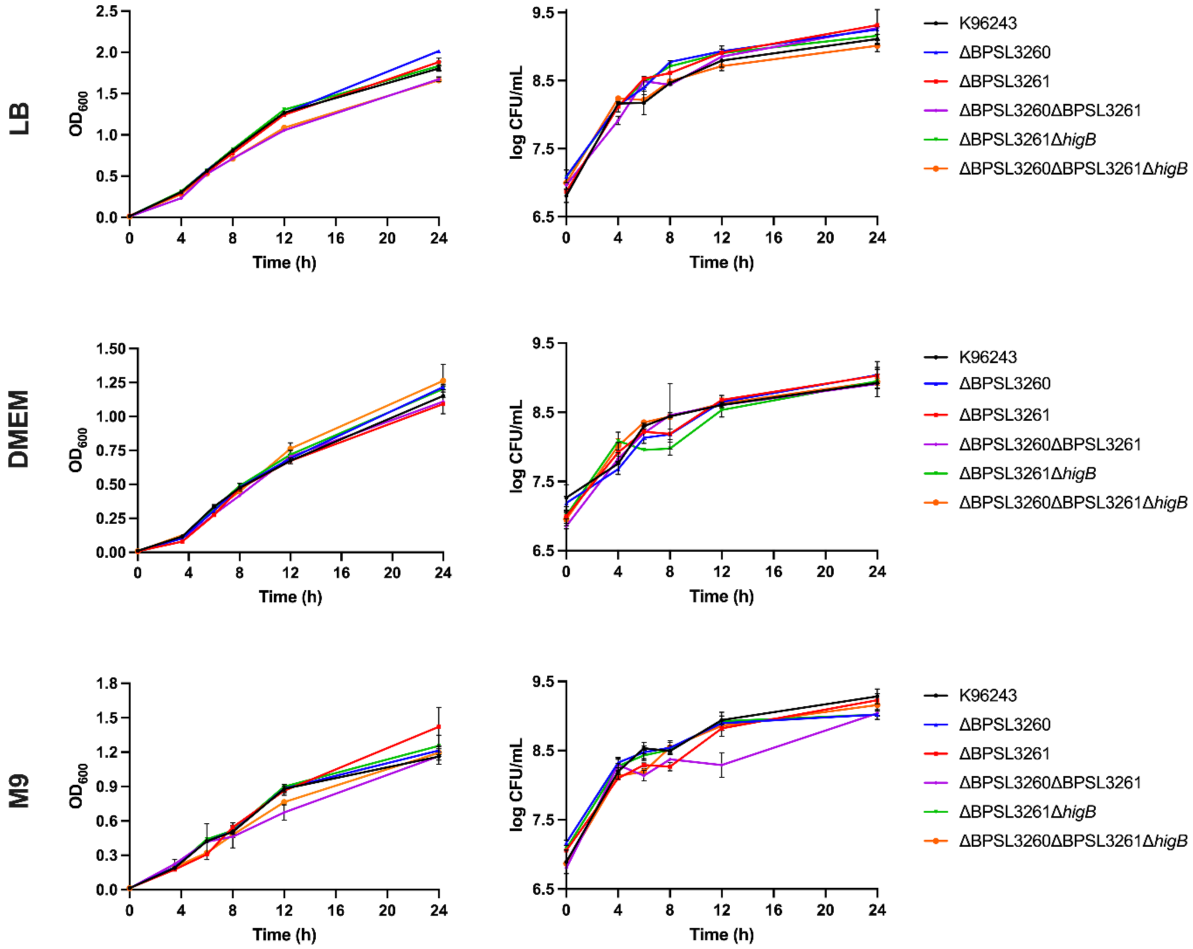

Figure S3. Growth curves. Growth curves in LB, minimal M9 media, and DMEM quantified by absorbance at OD_{600} and CFU enumeration plated into LB agar, from the WT and the different mutants. The experiments were performed in triplicate. Multiple *t*-test analysis was performed to evaluate significance.

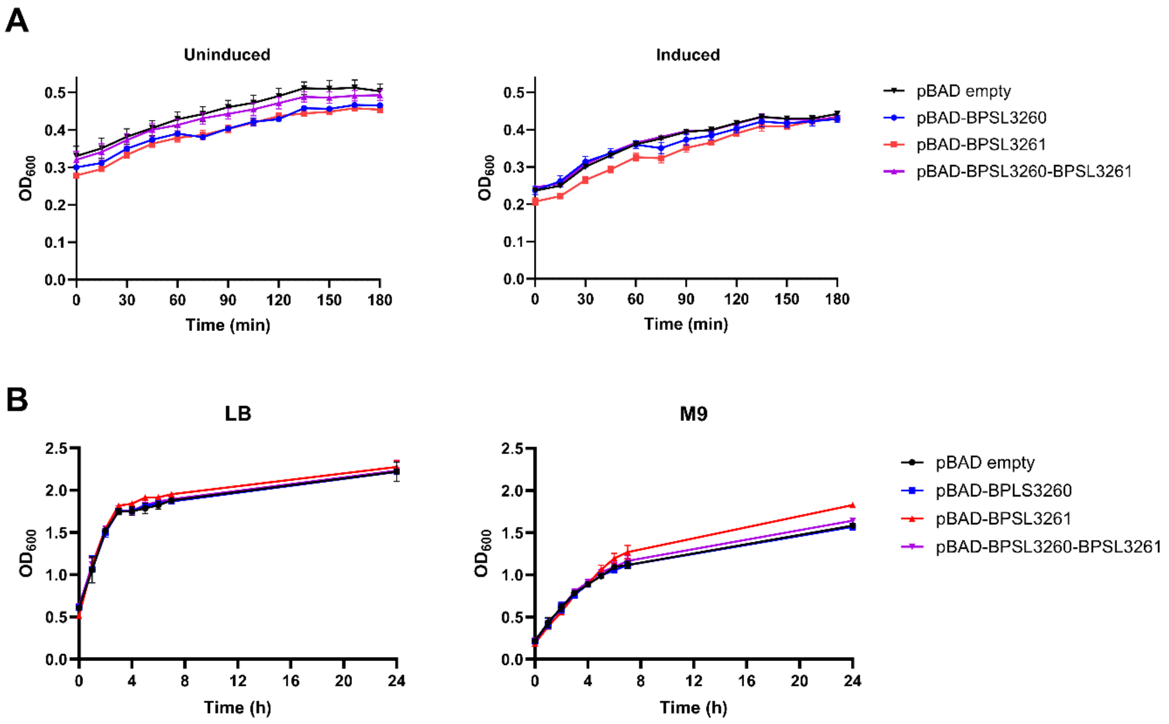

Figure S4. Growth curves from inducible system of antitoxin (BPSL3260), toxin (BPSL3261), or TA system (BPSL3260-BPSL3261) from *B. pseudomallei* in *E. coli*. No growing differences were seen in **(A)** short-time killing-rescue assay between induced or uninduced; not in **(B)** long-time overexpression at different media (LB: nutrient-rich; M9: minimal media).

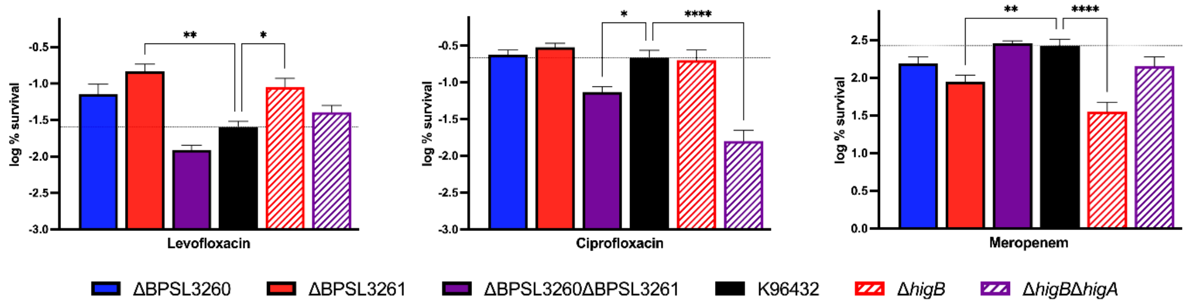

Figure S5. Supra-lethal antimicrobial treatment survival in two HigBA mutant systems. The same tendency was seen in the toxin mutants (ΔBPSL3261 and Δ*higB* -ΔBPSL3343-) in levofloxacin and meropenem treatment, as well as in the system mutants in ciprofloxacin (ΔBPLS3260ΔBPLS3261 and Δ*higB*Δ*higA* -ΔBPSL3343ΔBPS_RS18025-). Hence, those samples were submitted to RNA-seq. Dash lines indicate the wild type average in each condition. One-way ANOVA was performed to establish the significance in each group compared with the wild type; only significant differences are shown: * $p<0.05$; ** $p<0.01$; **** $p<0.0001$.

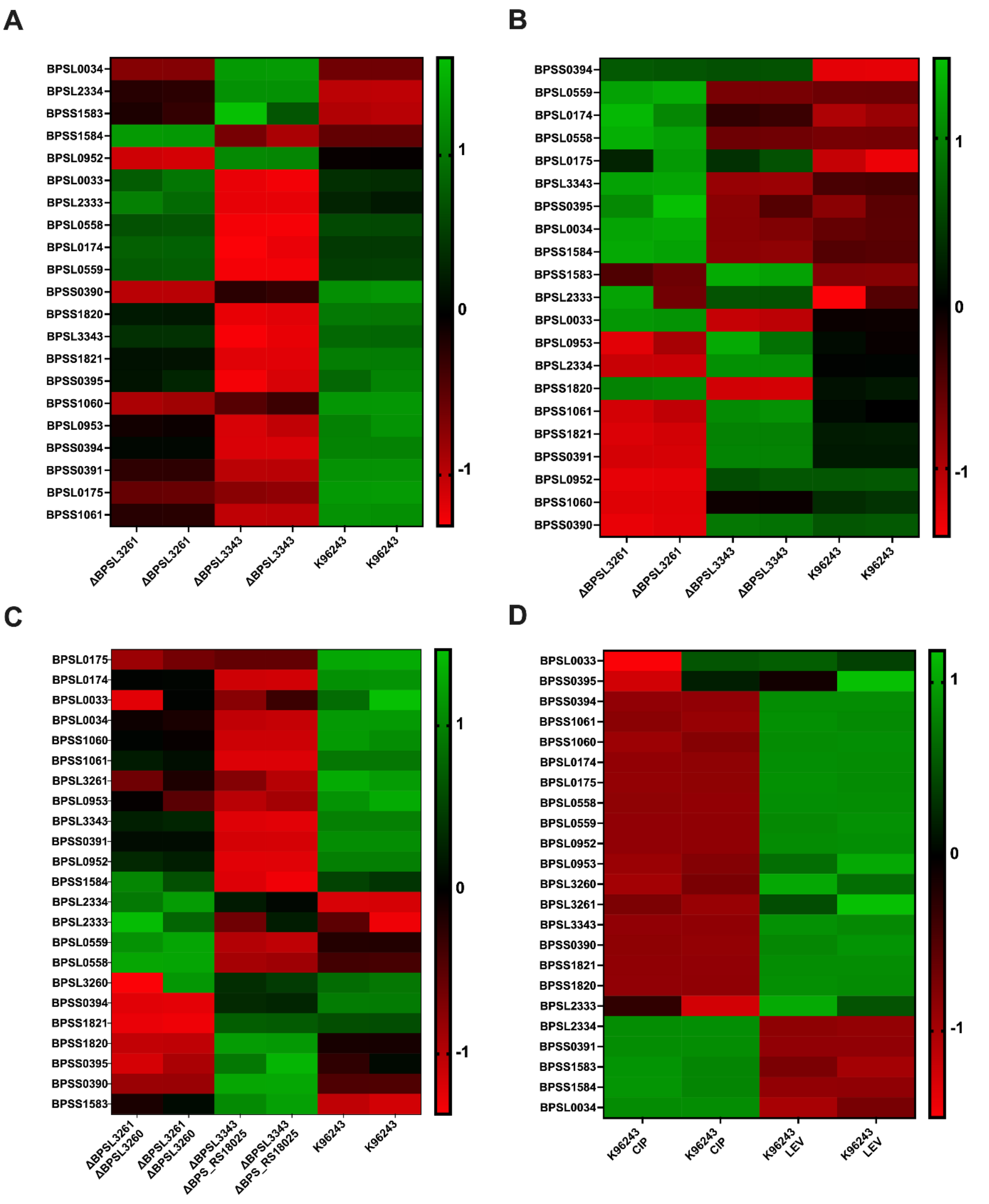

Figure S6. Heatmaps of only predicted TA systems genes from *B. pseudomallei* K96243, generated from RNA-seq data. Toxin mutants and systems mutants that exhibited similar behaviors under specific antibiotic treatments (Fig. S5) were submitted for RNA-sequencing in duplicate, and differential comparisons were established (Fig. 8). Panels show the expression

of manually selected previously predicted toxin and antitoxin genes when different strains were subjected to **A)** levofloxacin, **B)** meropenem, **C)** ciprofloxacin, or **D)** ciprofloxacin (CIP) and levofloxacin (LEV) in wild type. Along with the previously identified systems (9), the predicted new system BPSL1820-BPSL1821 obtained from RNA-seq (Fig. 8) was included. Genes nomenclature was obtained for all the panels from www.burkholderia.com database (last access, December 2023) for the reference strain K96243.

Table S1. Relation of the top one hundred most significant differential expressed genes shared among strains and conditions.

		CIP-LEV	LEV WT vs mutants	LEV mutant vs mutant	CIP WT vs mutants	CIP mutant vs mutant	MER WT vs mutants	MER mutant vs mutant
CIP-LEV	BPSS1525	■						
	BPSS0571	■						
	BPSL2191	■						
	BPSL1271	■		■				
	BPSL0470	■						
	BPSL0648	■		■				■
	BPSL3228	■				■		
	BPSL2159	■						
	BPSL2515	■				■		
	BPSL3318	■				■		
	BPSL2514	■						
	BPSL1212	■		■		■	■	
	BPSL3209	■				■	■	
	BPSS1062	■				■		
	BPSL3210	■						
	BPSL1509	■				■		
	BPSL1213	■		■				
	BPSL0649	■		■				■
	BPSL3213	■	■					
	BPSL0521	■		■				■
	BPSS0390	■	■			■		
	BPSL1511	■						■
	BPSL3344	■						
	BPSL3342	■				■	■	
	BPSL3345	■						
	BPSL2465	■						
	BPSL3204	■						
	BPSL0117	■		■		■		
	BPSL0174	■		■			■	
	BPSL2511	■						
	BPSL0356	■						■
	BPSL2298	■	■			■		
	BPSL0553	■		■				■
	BPSL3212	■	■					
	BPSS0860	■	■					
	BPSL0129	■						■
	BPSL0025	■		■				

	BPSS1063	■				■		
	BPSL0173	■		■				
	BPSL3211	■						
	BPSL1513	■				■		
	BPSL2512	■						
	BPSL1516	■						
	BPSL2188	■	■					
	BPSS1061	■						
	BPSL3118	■						
	BPSL2497	■						
	BPSL0280	■		■		■		
	BPSL1044	■		■		■		
	BPSL3041	■						
	BPSL3115	■						
	BPSL0176	■	■			■		
	BPSS1453	■						
	BPSL3403	■						
	BPSL3346	■						
	BPSL2749	■				■		
	BPSL0998	■						
	BPSL1515	■	■					
	BPSL1202	■		■				
	BPSL2299	■						
	BPSL0912	■						
	BPSL1514	■						
	BPSL3114	■						
	BPSL0552	■		■				
	BPSL0815	■						
	BPSL1244	■		■				
	BPSL2586	■				■		
	BPSL0172	■		■				
	BPSL1352	■						
	BPSS0277	■						
	BPSL1193	■	■			■	■	
	BPSL3117	■						
	BPSL0096	■						
	BPSL1508	■						
	BPSL0087	■		■				
	BPSL3426	■	■			■		
	BPSL3427	■				■		
	BPSL3339	■						
	BPSL1517	■						
	BPSL0124	■		■		■		■
	BPSS0404	■						
	BPSS1463	■						

	BPSL0171	■						
	BPSS0403	■						
	BPSS0625	■	■					
	BPSL3404	■						
	BPSS0405	■						
	BPSL0551	■						
	BPSL2499	■						
	BPSL0911	■	■	■		■		■
	BPSL2500	■						
	BPSL2498	■						
	BPSS0276	■				■		
	BPSS0394	■				■	■	
	BPSL0130	■	■					
	BPSL0170	■						
	BPSS0210	■						
	BPSL2323	■				■		
	BPSL3254A	■	■					
	BPSL2322	■						

		CIP-LEV	LEV WT vs mutants	LEV mutant vs mutant	CIP WT vs mutants	CIP mutant vs mutant	MER WT vs mutants	MER mutant vs mutant
**LEV WT vs Mutants**	BPSS1086		■					
	BPSL2531		■					
	BPSS1359		■					
	BPSS0127		■			■		
	BPSS0099		■			■		
	BPSL3071		■					
	BPSS1703		■					
	BPSL2385		■					
	BPSS1910		■					
	BPSS1723		■					
	BPSL2887		■					
	BPSL1830		■					
	BPSS0118		■					
	BPSS1296		■					
	BPSS0128		■					
	BPSL1953		■					
	BPSL2691		■					
	BPSL2825		■					
	BPSS1348		■					
	BPSS0094		■					
	BPSL2607		■					
	BPSL2177		■					

BPSL2892		■					
BPSL0437		■					
BPSS0449		■					
BPSS1163		■					
BPSL2022		■					
BPSL0450		■					
BPSS1311		■					
BPSL1782		■					
BPSL1434		■					
BPSS1726		■					
BPSL1783		■					
BPSS0726		■					
BPSL3367		■					
BPSS2312		■					
BPSL0194		■					
BPSL2052		■					
BPSL2027		■					
BPSS0032		■					
BPSS2250		■					
BPSL0915		■			■		■
BPSL2188	■	■					
BPSS1464		■			■		
BPSS1847		■					
BPSL0306		■					
BPSS0633		■					
BPSS0411		■					
BPSL1203		■				■	
BPSS0862		■					
BPSS1838		■					
BPSS1837		■					
BPSS2086		■					
BPSL0587		■		■			
BPSL2466		■					
BPSS1815		■					
BPSS1995		■					
BPSL1515	■	■					
BPSS0402		■					
BPSL0752		■					
BPSL3212	■	■					
BPSL0494		■					
BPSL0643		■					
BPSS0860	■	■					
BPSL0916		■					■
BPSL1580		■					
BPSL2988		■			■		

		CIP-LEV	LEV WT vs mutants	LEV mutant vs mutant	CIP WT vs mutants	CIP mutant vs mutant	MER WT vs mutants	MER mutant vs mutant
	BPSS0880		■					
	BPSS0354		■					
	BPSL3350		■					
	BPSL1912		■					
	BPSL3213	■	■					
	BPSL0177		■					
	BPSS0625	■	■					
	BPSS1839		■					
	BPSS0390	■	■			■		
	BPSL0131		■					
	BPSS0398		■					
	BPSL0894		■					
	BPSS0987		■					
	BPSL0130	■	■					
	BPSL1074		■					
	BPSS1994		■					
	BPSL3426	■	■			■		
	BPSS2108		■					
	BPSS2090		■					
	BPSL1073		■		■			
	BPSS1065		■					
	BPSS2089		■					
	BPSL0176	■	■			■		
	BPSS1060		■					
	BPSL2298	■	■			■		
	BPSS2087		■					
	BPSL0175		■					
	BPSL0911	■	■	■		■		■
	BPSL1304A		■					
	BPSS2091		■					
	BPSL1193	■	■			■	■	
	BPSL3254A	■	■					
	BPSL3116		■					
**LEV Mutant vs Mutant**	BPSL0020			■				
	BPSL0811			■				
	BPSL0556			■				
	BPSL0093			■			■	
	BPSL0559			■				
	BPSL0458a			■				
	BPSL1009			■				

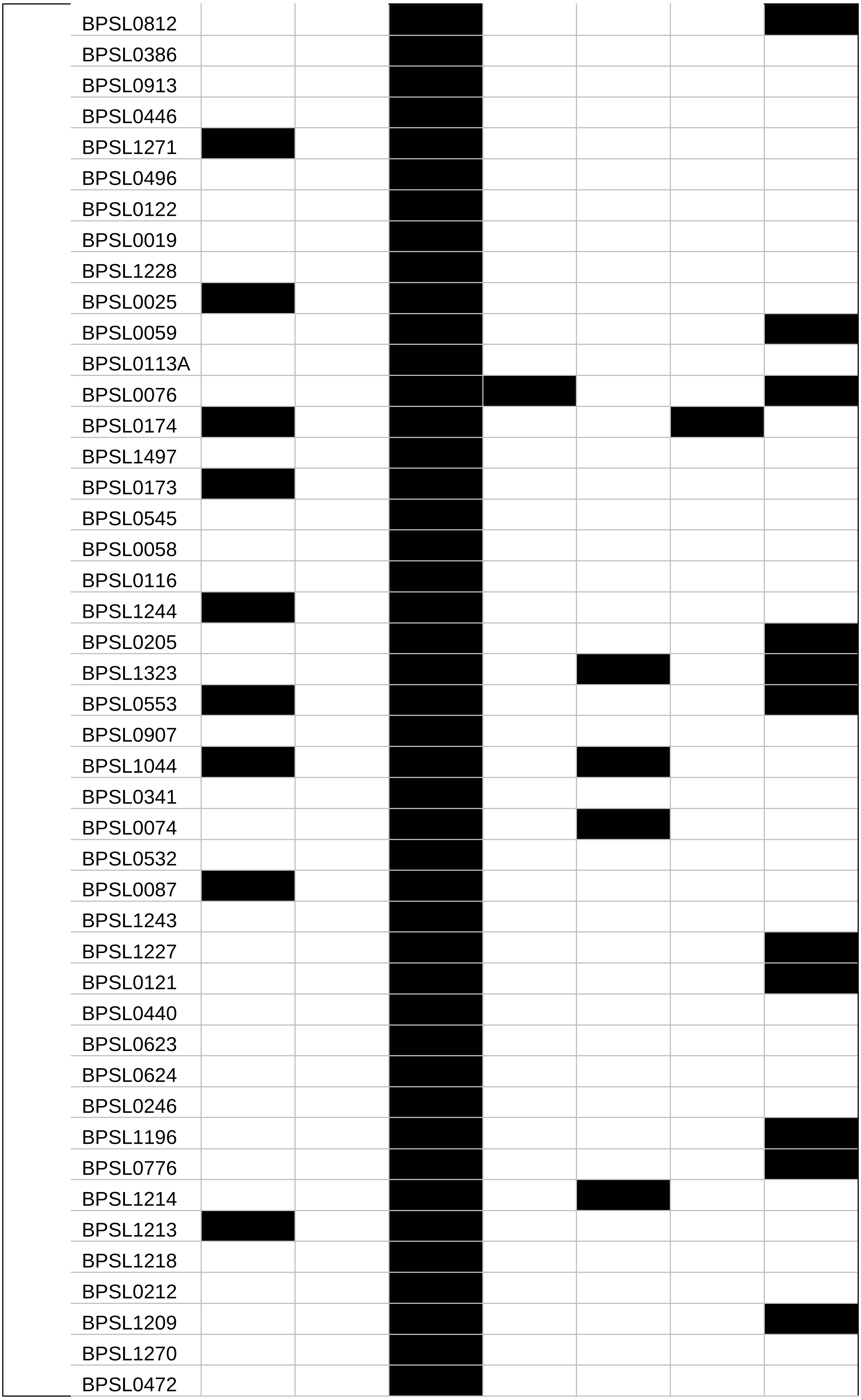
BPSL0812
BPSL0386
BPSL0913
BPSL0446
BPSL1271
BPSL0496
BPSL0122
BPSL0019
BPSL1228
BPSL0025
BPSL0059
BPSL0113A
BPSL0076
BPSL0174
BPSL1497
BPSL0173
BPSL0545
BPSL0058
BPSL0116
BPSL1244
BPSL0205
BPSL1323
BPSL0553
BPSL0907
BPSL1044
BPSL0341
BPSL0074
BPSL0532
BPSL0087
BPSL1243
BPSL1227
BPSL0121
BPSL0440
BPSL0623
BPSL0624
BPSL0246
BPSL1196
BPSL0776
BPSL1214
BPSL1213
BPSL1218
BPSL0212
BPSL1209
BPSL1270
BPSL0472

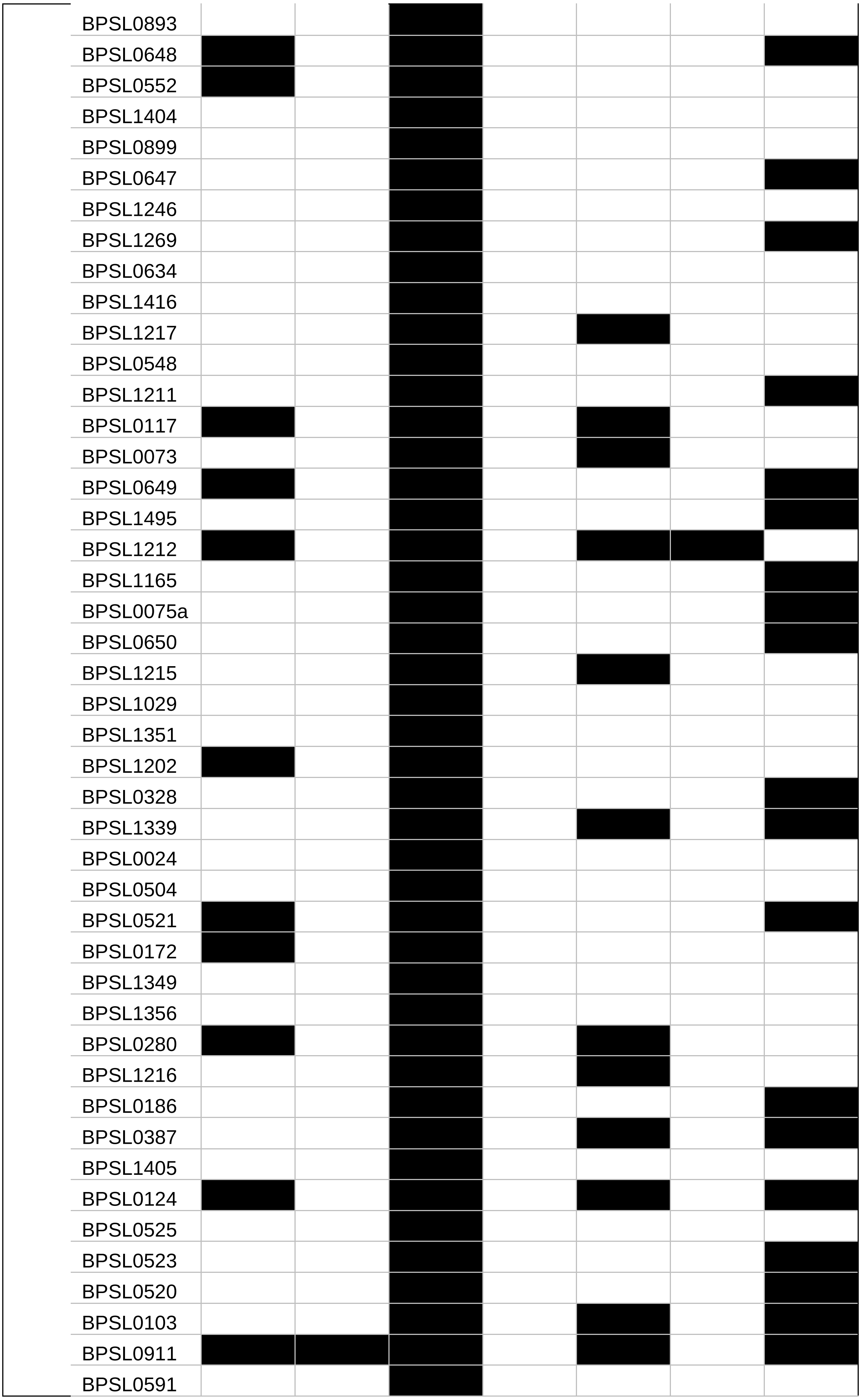
BPSL0893
BPSL0648
BPSL0552
BPSL1404
BPSL0899
BPSL0647
BPSL1246
BPSL1269
BPSL0634
BPSL1416
BPSL1217
BPSL0548
BPSL1211
BPSL0117
BPSL0073
BPSL0649
BPSL1495
BPSL1212
BPSL1165
BPSL0075a
BPSL0650
BPSL1215
BPSL1029
BPSL1351
BPSL1202
BPSL0328
BPSL1339
BPSL0024
BPSL0504
BPSL0521
BPSL0172
BPSL1349
BPSL1356
BPSL0280
BPSL1216
BPSL0186
BPSL0387
BPSL1405
BPSL0124
BPSL0525
BPSL0523
BPSL0520
BPSL0103
BPSL0911
BPSL0591

BPSL0597			■				■
BPSL0590			■				
BPSL0605			■				

		CIP-LEV	LEV WT vs mutants	LEV mutant vs mutant	CIP WT vs mutants	CIP mutant vs mutant	MER WT vs mutants	MER mutant vs mutant
**CIP WT vs Mutants**	BPSL3396				■			
	BPSL0202				■			
	BPSL1828				■			
	BPSL3398				■			
	BPSL1929				■			
	BPSL0004				■			■
	BPSL0460				■			
	BPSL0696				■		■	
	BPSL3191				■			
	BPSL3395				■			
	BPSL0972				■		■	
	BPSL2982				■			
	BPSL2386				■			
	BPSL3069				■			
	BPSL3216				■			
	BPSL3073				■			
	BPSL2550				■			
	BPSL3378				■			
	BPSL3080				■			
	BPSL0076			■	■			■
	BPSL3152				■			
	BPSL3051				■			
	BPSL2765				■			
	BPSL2439				■			
	BPSL2613				■			
	BPSL1982				■			
	BPSL3068				■			
	BPSL2925				■			
	BPSL2165				■			
	BPSL2992				■			
	BPSL3362				■			
	BPSS1763				■			
	BPSL2344				■			
	BPSL2701				■			
	BPSL2564				■			
	BPSL1803				■			
	BPSL0082				■			

BPSL0279							
BPSL2440							
BPSL0443							
BPSL1845							
BPSL2612							
BPSL2155							
BPSL3231							
BPSL2523							
BPSL1367							
BPSL0381							
BPSL1402							
BPSS0280							
BPSS0057							
BPSL1841							
BPSL1839							
BPSL2986							
BPSL2563							
BPSL3390							
BPSL3067							
BPSL2565							
BPSL1966							
BPSL2266							
BPSL0501							
BPSL2473							
BPSL3040							
BPSL2171							
BPSL2614							
BPSL2525							
BPSL1591							
BPSL3052							
BPSL1233							
BPSL1403							
BPSL2747							
BPSS0222							
BPSL1980							
BPSL2522							
BPSS1727							
BPSS1871							
BPSS0065							
BPSS0203							
BPSL1073							
BPSS2065							
BPSL0619							
BPSS2053							
BPSS0159							

		CIP-LEV	LEV WT vs mutants	LEV mutant vs mutant	CIP WT vs mutants	CIP mutant vs mutant	MER WT vs mutants	MER mutant vs mutant
	BPSS1272				■			
	BPSL2185				■			
	BPSL1660				■			
	BPSS2192				■			
	BPSS0524				■			
	BPSL2477				■			
	BPSS2051				■			
	BPSS2188				■			
	BPSS2048				■			
	BPSS1391				■			
	BPSS2045				■			
	BPSS1495				■			
	BPSL0587		■		■			
	BPSS2063				■			
	BPSS1519				■			
	BPSS0251				■			
	BPSS1521				■			
	BPSS2041				■			
**CIP Mutant vs Mutant**	BPSS1062	■				■		
	BPSS0099		■			■		
	BPSS1063	■				■		
	BPSS0123					■		
	BPSS0091					■		
	BPSS0098					■		
	BPSS0119					■		
	BPSS0121					■		
	BPSS0087					■		
	BPSS0122					■		
	BPSS0126					■		
	BPSS0016					■		
	BPSS0127		■			■		
	BPSS0058					■		
	BPSS0018					■		
	BPSL2803					■		
	BPSL1743					■		■
	BPSL0073			■		■		
	BPSS1602					■		
	BPSS1718					■		
	BPSL2272					■		
	BPSS0637					■		

	BPSL2837					■		
	BPSL3381					■		
	BPSL0103			■		■		■
	BPSL2535					■		
	BPSS1149					■		
	BPSL3380					■		
	BPSL2423					■		
	BPSL2301					■		
	BPSL1217			■		■		
	BPSL1339			■		■		■
	BPSL0074			■		■		
	BPSL1323			■		■		■
	BPSS1356					■		
	BPSL3221					■		
	BPSL1216			■		■		
	BPSL3318	■				■		
	BPSL1215			■		■		
	BPSL1212	■		■		■	■	
	BPSL2129					■		
	BPSL2516					■		
	BPSS1755					■		
	BPSL2697					■		
	BPSL1214			■		■		
	BPSL2521					■		
	BPSL3228	■				■		
	BPSL1513	■				■		
	BPSS0391					■		
	BPSL2515	■				■		
	BPSL3342	■				■	■	
	BPSS1783					■		
	BPSS0081					■		
	BPSS0394	■				■	■	
	BPSL3193					■		
	BPSL2298	■	■			■		
	BPSL0124	■		■		■		■
	BPSL2444					■		
	BPSL1509	■				■		
	BPSL0280	■		■		■		
	BPSL0387			■		■		■
	BPSL0911	■	■	■		■		■
	BPSL2749	■				■		
	BPSS1748					■		
	BPSL3209	■				■	■	
	BPSL1925					■		
	BPSL1044	■		■		■		

		CIP-LEV	LEV WT vs mutants	LEV mutant vs mutant	CIP WT vs mutants	CIP mutant vs mutant	MER WT vs mutants	MER mutant vs mutant
	BPSL3196					■		
	BPSL1046					■		
	BPSS0276	■				■		
	BPSL0638					■		■
	BPSS1821					■		
	BPSS1819					■	■	
	BPSS1784					■		
	BPSS1464		■			■		
	BPSS1820					■		
	BPSL2297					■		
	BPSL1942					■		
	BPSL0117	■		■		■		
	BPSL3217					■		
	BPSL3178					■	■	
	BPSL2988		■			■		
	BPSS1817					■		
	BPSL0176	■	■			■		
	BPSL3351					■		
	BPSL1943					■		
	BPSL1461					■		■
	BPSL3194					■		
	BPSL0915		■			■		■
	BPSL3352					■		
	BPSL2586	■				■		
	BPSL2323	■				■		
	BPSL1376					■		
	BPSL3427	■				■		
	BPSL1193	■	■			■	■	
	BPSL2989					■		
	BPSS0390	■	■			■		
	BPSL2585					■		
	BPSL3197					■		
	BPSL3426	■	■			■		
**MER WT vs Mutants**	BPSS0394	■				■	■	
	BPSL1408						■	
	BPSL0174	■		■			■	
	BPSL0090						■	
	BPSL0301						■	
	BPSS1489						■	
	BPSS0279						■	

	BPSL2795						■	
	BPSL1644						■	
	BPSS0362						■	
	BPSS1448						■	
	BPSS2028						■	
	BPSL3304						■	
	BPSL2196						■	
	BPSS0438						■	
	BPSL1643						■	
	BPSL0361						■	
	BPSS1337						■	
	BPSL1193	■	■			■	■	
	BPSL0793						■	
	BPSL0093			■			■	
	BPSL2016						■	
	BPSL1885						■	
	BPSS1227						■	
	BPSS0369						■	
	BPSL2108						■	
	BPSS1386						■	
	BPSS0317						■	
	BPSL1679						■	
	BPSL3430						■	
	BPSL2866						■	
	BPSL0542						■	
	BPSL2163						■	
	BPSS1709						■	
	BPSL0972				■		■	
	BPSL1447						■	
	BPSL1212	■		■		■	■	
	BPSL1558						■	
	BPSS0410						■	
	BPSL1539						■	
	BPSS0078						■	
	BPSL3428						■	
	BPSL2288						■	
	BPSL0696				■		■	
	BPSS0708						■	
	BPSL1190						■	
	BPSL2872						■	
	BPSL0963						■	
	BPSL3342	■				■	■	
	BPSL3279						■	
	BPSS1819					■	■	
	BPSS1934						■	

	BPSL1004							
	BPSL3332							
	BPSL1350							
	BPSL3178							
	BPSL0778							
	BPSL1020							
	BPSL1914							
	BPSL1469							
	BPSS1635							
	BPSL0904							
	BPSL3214							
	BPSL2380							
	BPSS2302							
	BPSL0432							
	BPSL3209							
	BPSL3074							
	BPSL3323							
	BPSL0318							
	BPSL2262							
	BPSL0357							
	BPSS2283							
	BPSL0873							
	BPSL0199							
	BPSL0928							
	BPSL3236							
	BPSL0299							
	BPSL1277							
	BPSL3258							
	BPSS1327							
	BPSL3365							
	BPSS1485							
	BPSS1936							
	BPSS1713							
	BPSL2903							
	BPSS1932							
	BPSL1917							
	BPSL1203							
	BPSL3127							
	BPSS1824							
	BPSL2668							
	BPSL0181							
	BPSL1417							
	BPSS1553							
	BPSS0927							
	BPSS1236							

	BPSL2484						■	
	BPSL3423						■	
	BPSS1141						■	

		CIP-LEV	LEV WT vs mutants	LEV mutant vs mutant	CIP WT vs mutants	CIP mutant vs mutant	MER WT vs mutants	MER mutant vs mutant
**MER Mutant vs Mutant**	BPSL1617							■
	BPSL1622							■
	BPSL0483							■
	BPSL1721							■
	BPSL1623							■
	BPSL1549							■
	BPSL0493							■
	BPSL1591				■			■
	BPSL0324							■
	BPSL0597			■				■
	BPSL0419							■
	BPSL0874							■
	BPSL1081							■
	BPSL0059			■				■
	BPSL0356	■						■
	BPSL1534							■
	BPSL0249							■
	BPSL1567							■
	BPSL1083							■
	BPSL1137							■
	BPSL1413							■
	BPSL1535							■
	BPSL1222							■
	BPSL1445							■
	BPSL0638					■		■
	BPSL1498							■
	BPSL1227			■				■
	BPSL1196			■				■
	BPSL1188							■
	BPSL0502							■
	BPSL0004				■			■
	BPSL1269			■				■
	BPSL0098							■
	BPSL0911	■	■	■		■		■
	BPSL1495			■				■
	BPSL1460							■
	BPSL0898							■

BPSL0648	■		■				■
BPSL1743					■		■
BPSL1211			■				■
BPSL1323			■		■		■
BPSL0649	■		■				■
BPSL1461					■		■
BPSL0394							■
BPSL0124	■		■		■		■
BPSL0918							■
BPSL1165			■				■
BPSL1548							■
BPSL0186			■				■
BPSL1206							■
BPSL0241							■
BPSL1528							■
BPSL1484							■
BPSL0203							■
BPSL0001							■
BPSL0870							■
BPSL0205			■				■
BPSL0553	■		■				■
BPSL0520			■				■
BPSL0444							■
BPSL0206							■
BPSL0103			■		■		■
BPSL1511	■						■
BPSL0939							■
BPSL0121			■				■
BPSL0076			■	■			■
BPSL0647			■				■
BPSL1339			■		■		■
BPSL1458							■
BPSL0776			■				■
BPSL0734							■
BPSL1631							■
BPSL1355							■
BPSL1510							■
BPSL0812			■				■
BPSL0426							■
BPSL0650			■				■
BPSL0240							■
BPSL0328			■				■
BPSL1120							■
BPSL1359							■
BPSL0871							■

	BPSL1311							
	BPSL1209							
	BPSL1744							
	BPSL0075a							
	BPSL1015							
	BPSL0425							
	BPSL1059							
	BPSL0204							
	BPSL0521							
	BPSL0915							
	BPSL0916							
	BPSL0387							
	BPSL0523							
	BPSL0129							
	BPSL0735							
	BPSL1491							
	BPSL0336							
	BPSL0937							
